# Efficient tagging of endogenous proteins in human cell lines for structural studies by single-particle cryo-EM

**DOI:** 10.1073/pnas.2302471120

**Published:** 2023-07-24

**Authors:** Wooyoung Choi, Hao Wu, Klaus Yserentant, Bo Huang, Yifan Cheng

**Affiliations:** ^a^Department of Biochemistry and Biophysics, University of California, San Francisco, CA 94143; ^b^Department of Pharmaceutical Chemistry, University of California, San Francisco, CA 94143; ^c^Chan Zuckerberg Biohub-San Francisco, San Francisco, CA 94158; ^d^HHMI, University of California, San Francisco, CA 94143

**Keywords:** CRISPR tagging, endogenous protein, human cell lines, cryo-EM, structure

## Abstract

CRISPR/Cas9-based genome engineering has revolutionized our ability to manipulate biological systems, particularly in higher organisms. Here, we designed a set of homology-directed repair donor templates that enable efficient tagging of endogenous proteins with affinity tags by transient transfection and selection of genome-edited cells in various human cell lines. Combined with technological advancements in single-particle cryogenic electron microscopy, this strategy allows efficient structural studies of endogenous proteins captured in their native cellular environment and during different cellular processes. We demonstrated this strategy by tagging six different human proteins in both HEK293T and Jurkat cells. Moreover, analysis of endogenous glyceraldehyde 3-phosphate dehydrogenase (GAPDH) in HEK293T cells allowed us to follow its behavior spatially and temporally in response to prolonged oxidative stress, correlating the increased number of oxidation-induced inactive catalytic sites in GAPDH with its translocation from cytosol to nucleus.

## Significance

We present a versatile CRISPR/Cas strategy to insert affinity tags to endogenous proteins in human cell lines and demonstrated its efficiency by tagging human proteins in both HEK293T and Jurkat cells. Such efficient tagging extends the boundary of structural studies of human endogenous proteins. By tagging endogenous GAPDH in HEK293T cells, we demonstrate tracking its temporal and spatial structural changes in response to prolonged oxidative stress.

Modern biology, structural biology in particular, has benefitted hugely from the recombinant technology that allows plasmid expression of target genes in a heterologous system. Different expression systems, such as bacterial (*Escherichia coli*) (1), insect (Sf9) (2), yeast (3, 4), and mammalian [e.g., HEK (5) or CHO (6)] cells, are often used to overexpress proteins of interest from different species or origins. They have enabled efficient production of high-quality and homogenous samples with quantity sufficient for structural characterization. One advantage of the heterologous expression is the ability to incorporate various affinity tags for protein purification. It also enables efficient protein manipulations, such as introducing point mutations or domain deletions, for dissecting protein functions (7, 8). However, producing functional large, dynamic, and multisubunits protein complexes that are folded and assembled properly remains challenging and often requires significant trial-and-error efforts (9).

Nowadays, with single-particle cryogenic electron microscopy (cryo-EM) as a mainstream technology in structural biology (10), analysis of endogenous protein complexes assembled in the native environment has regained attractiveness. The requirement of both quantity and purity of any target sample needed for structural analysis is much less stringent than used to be. Powerful computational particle classification algorithms (11–13) further loosen the requirement of sample homogeneity. Capturing endogenous proteins for structural characterization thus becomes routinely feasible. It further offers opportunities to monitor complexes at various stages during their assembly, or changes under different cellular environments, such as under various stresses (14–19).

A remaining major challenge in studying mammalian endogenous proteins is their purification, which often requires sophisticated approaches that are designed and optimized for individual targets (18, 20). The development of genome editing technology making use of CRISPR/Cas (21–24) provides tools to overcome this challenge by genetically incorporating affinity tags to targeted genes in cells, thus enabling affinity purification of endogenous proteins from various cell lines (15, 25, 26).

Here, we present a versatile strategy of tagging endogenous proteins in human cell lines. The strategy is implemented by using a multicomponent homology-directed repair (HDR) donor template that can be easily converted to all three commonly used HDR donor forms for parallelized knock-in experiments. Using this strategy, we successfully tagged six proteins with diverse endogenous expression levels and subcellular locations in both HEK293T and Jurkat cells, and we purified them from HEK293T cells for structural studies by single-particle cryo-EM. As a further proof of concept of tagging other human cell lines, we successfully tagged two of these targets in a breast cancer cell line MDA-MB468. Focusing on endogenous human glyceraldehyde-3-phosphate dehydrogenase (GAPDH), we unravel additional structural and mechanistic insights that are not revealed by using recombinant proteins. While our work focuses on structural characterization of endogenous human proteins, the same strategy can be applied to other types of cell lines and used beyond structural biology.

## Results

### Designing a Convertible Multicomponent HDR Template.

**Fig. 1.**
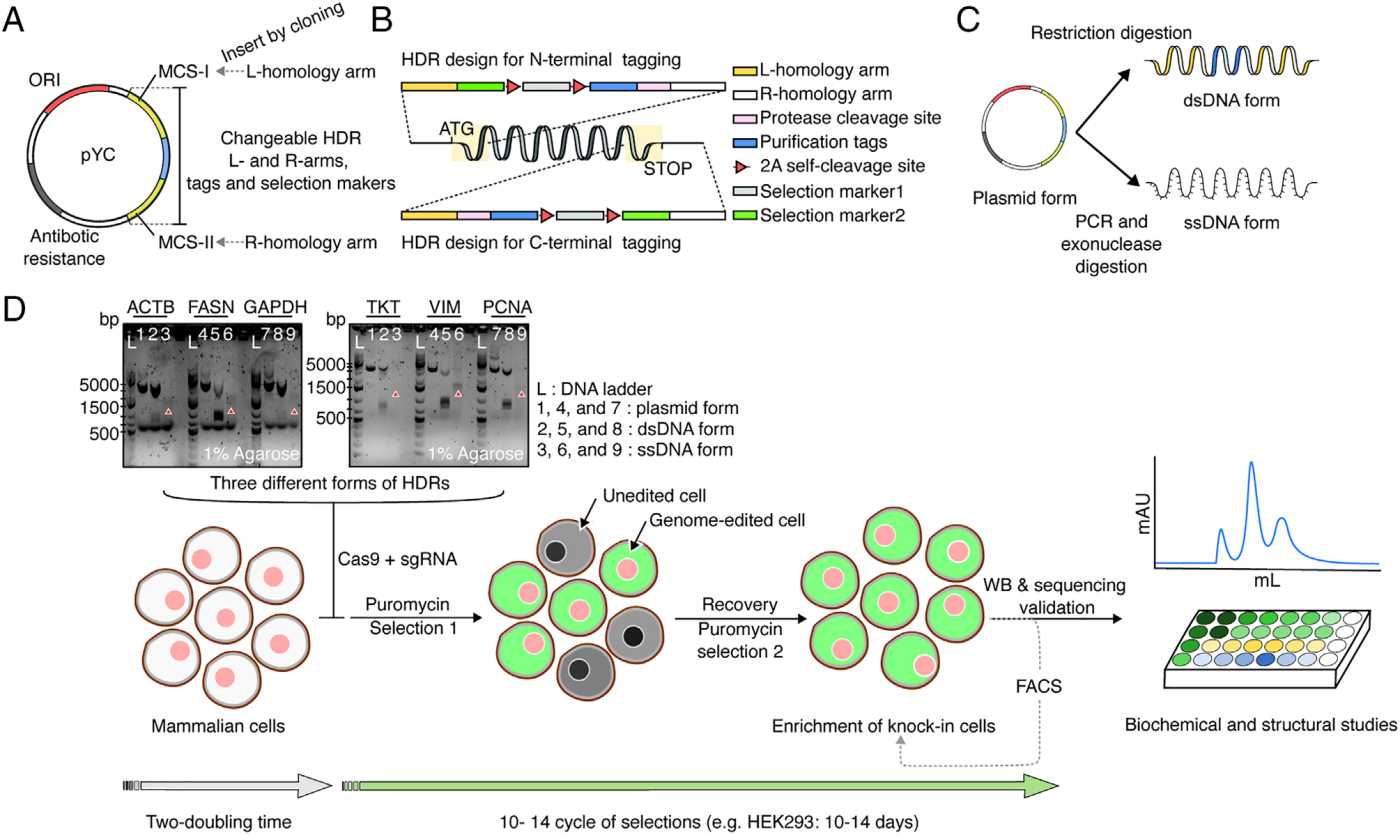
HDR donor template design and the endogenous protein tagging scheme. (*A*) Schematic of the pYC plasmid design. All elements are colored and labeled. In addition to the changeable HDR donor, which contains the changeable tags and selection markers (blue) flanked by corresponding left and right homology arms (yellow), the plasmid contains ColE1 origin of replication (red) and ampicillin resistance gene (gray) for routine amplification in bacteria. (*B*) The changeable HDR donor to tag the N terminus (above) or C terminus (below) of a target protein (middle). The affinity tags and selection markers (antibiotic selection and fluorescence) are integrated immediately after the start codon (for N-terminal tagging) or before the stop codon (for C-terminal tagging). Two P2A self-cleavage sites (red triangle) are located between two reporter markers and between the gene of interest and the adjacent selection marker. (*C*) Workflow of converting a plasmid into dsDNA and ssDNA HDR donors. (*D*) Agarose gels with SYBR gold show size of DNA corresponding to all forms of HDR donors. The generated HDR donor is transfected into the mammalian cells together with the corresponding sgRNA containing plasmid, px458. Successfully edited cells are selected by puromycin treatment and can be further selected by FACS if necessary (dotted line). Knock-ins are validated by western blot and cDNA sequencing after mRNA extraction and reverse transcription in vitro. The validated knock-in cells are used in subsequent biological experiments.

To facilitate efficient tagging by CRISPR/Cas9, we generated plasmid Yifan Cheng with different variations (pYC) (Fig. 1*A* and *SI Appendix,* Fig. S1). They contain a DNA fragment that we would like to insert at the targeted locus, flanked by two multiple cloning sites (MCS) for insertion of left and right homology arms (L- and R-arms) flanking the targeted locus. The plasmid also contains a standard ColE1 origin of replication and antibiotic resistance β-lactamase for efficient amplification in *Escherichia coli*. The inserted DNA fragment between two homology arms contains affinity tags, whose choice can vary to match specific experimental goals, and selection markers (Fig. 1*B*). For target protein purification, any commonly used affinity tags, such as 3×FLAG, twin-strep, or ALFA-tag, can be inserted individually or in combination on either the N or C terminus of a target protein (*SI Appendix,* Fig. S1*A*). A protease cleavage site is introduced between the tag and the targeted gene, allowing cleavage of the purification tag when necessary. For efficient selection of genome-edited cells, we use two selection markers, a mammalian antibiotic resistance marker, such as puromycin N-acetyl transferase, and a fluorescent marker, such as mNeonGreen. A P2A self-cleaving peptide (27) is inserted between the affinity tag and the selection marker, as well as between the two selection markers. In addition, this template can be easily converted into other forms for different purposes, such as for cellular localization of a target protein by fluorescence microscopy or to enable fluorescence size exclusion chromatography (discussed in the following). Variations of the template are summarized in *SI Appendix,* Fig. S1.

Currently, there are three forms of HDR donors that are commonly used for knock-in approaches, i.e., plasmid, double-stranded and single-stranded DNA (dsDNA and ssDNA). Among them, ssDNA is considered a promising HDR donor form, due to its higher efficiency and lower off-target rates (28–31), though the plasmid and dsDNA donors are easier to handle for larger cargos. Our previous work used ssDNA donors combined with split fluorescent protein (FP) for cell selection (26). The pYC template, on the other hand, is designed to be compatible with all three HDR donor forms. The two MCSs in the plasmid can be used as cleavage sites to convert the plasmid HDR donor into a dsDNA HDR donor. With a pair of primers, in which the 3′ primer contains a phosphorylation modification at the 5′ end, and use of exonuclease digestion, a ssDNA HDR donor can be generated from the respective dsDNA form (Fig. 1*C*). This versatility is practically helpful as the editing efficiency and fidelity of different HDR donor forms vary from gene to gene, and even among different cell types (32, 33).

Once inserted at the target locus, this design produces three proteins: the target protein with the affinity tags and two selection markers separated from the targeted protein. Genome-edited cells are selected in two steps, i.e., antibiotic treatment followed by fluorescence-activated cell sorting (FACS). Because the life spans of the selection markers are independent from the target protein, their accumulation allow for efficient selection of edited cells even if the target protein has a short life span or low endogenous expression level. In practice, the majority of unedited cells are removed by puromycin treatment. At this point, the knock-in result can be checked by western blot, sequencing, and/or fluorescence light microscopy. The enriched population of genome-edited cells makes the final sorting by FACS more efficient or even unnecessary. This procedure is illustrated in Fig. 1*D*. A major advantage of this approach is that the success rate of generating genome-edited cell lines is less dependent on the initial efficiency of CRISRP/Cas9 knock-in.

### Tagging of Endogenous Proteins in HEK293T, Jurkat, and MDA-MB468 Cells.

We applied the pYC-3×FLAG template described above to insert in both HEK293T and Jurkat cells a 3×FLAG tag to six different protein targets: β-actin (ACTB, 42 kDa), fatty acid synthase (FASN, 273 kDa), glyceraldehyde 3-phosphate dehydrogenase (GAPDH, 36 kDa), transketolase (TKT, 68 kDa), vimentin (VIM, 54 kDa), and proliferating cell nuclear antigen (PCNA, 29 kDa). Correlated with their biological and cellular functions, each of these proteins has different cellular expression levels (34, 35). For each target, we generated three forms of HDR donor, i.e., plasmid, dsDNA, and ssDNA (Fig. 1*D*, DNA gel panel).

**Fig. 2.**
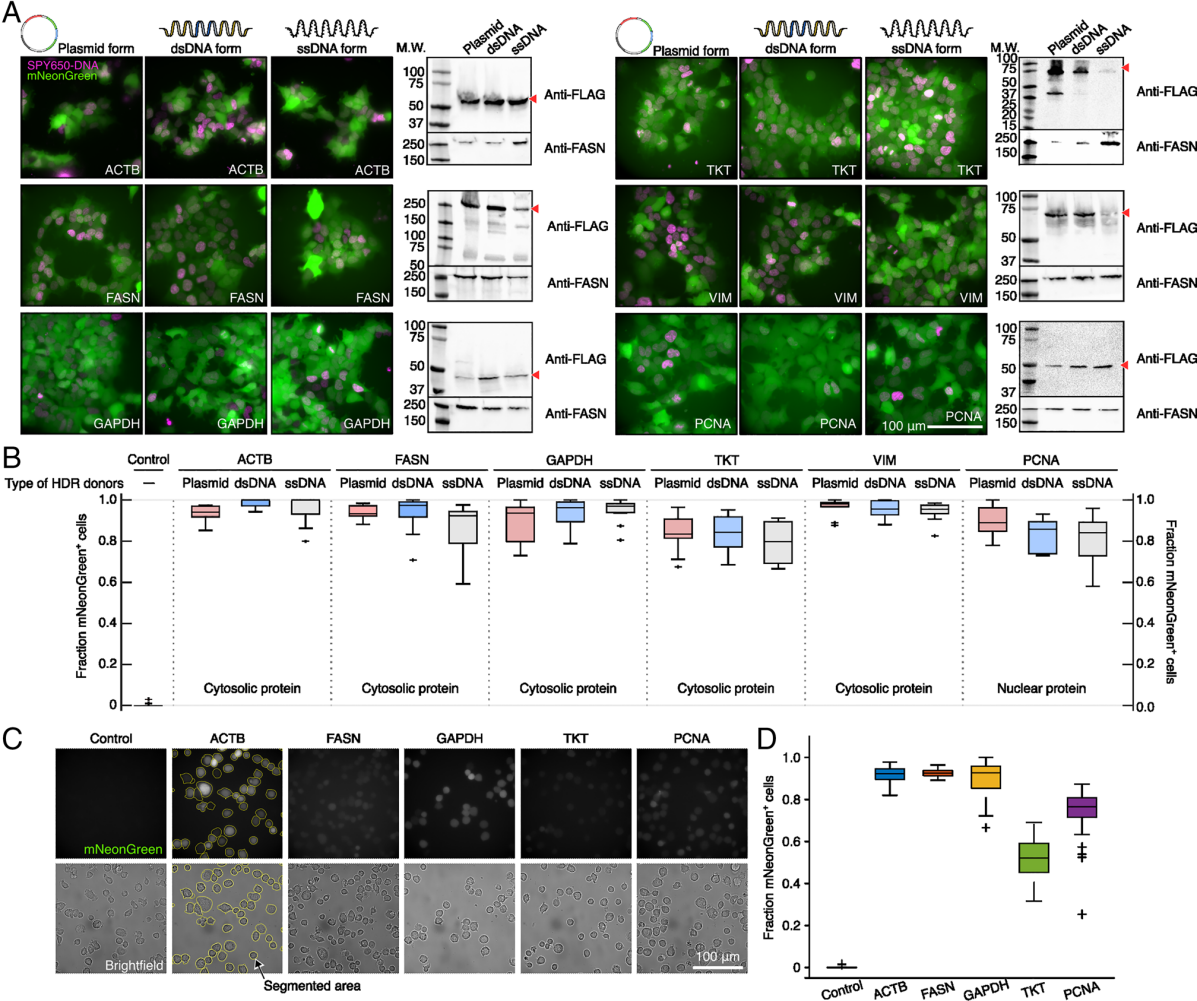
Tagging endogenous proteins in HEK293T and Jurkat cells. (*A*) Fluorescence microscopy images (*Left*) and anti-FLAG western blot (*Right*) of edited HEK293T cells after puromycin treatment. Six genes, *ACTB, FASN*, *GAPDH*, *TKT, VIM*, and *PCNA*, are tagged by CRISPR/Cas9 using indicated HDR donors. To segment cells, the noncovalent DNA stain SPY650-DNA is applied. For western blots, anti-FLAG antibody is used for validating the target protein while anti-FASN antibody serves as loading control. The relevant bands are highlighted with red triangles. (*B*) Knock-in efficiency for each HDR donor type across the six target genes was measured as the fraction of mNeonGreen-positive (mNeonGreen^+^) cells. Knock-in experiments and validation processes were repeated three times. (*C*) The fluorescence images of Jurkat cells after knock-in resulting expression of mNeonGreen from five different loci (upper row). Bright-field images (bottom row) are used to segment cells for automated mNeonGreen intensity extraction (yellow dotted lines). (*D*) Knock-in efficiency across the five different genes in Jurkat cells measured as fraction of mNeonGreen positive cells. The knock-in experiments and validation processes were repeated twice in Jurkat cells.

For HEK293T cells, we first transiently transfected the adherent cells with one form of HDR donor and the px458 plasmid to express Cas9 and the corresponding sgRNA in cells. After puromycin treatment, we performed immunoblotting to evaluate the outcomes of the tagging procedures using different HDR donors (Fig. 2*A*). We succeeded in tagging all six targets (Fig. 2 *A* and *B*), among which TKT has presumably the lowest endogenous expression level in HEK293T cells (34, 35). Since the fluorescent signal generated from FPs encoded on the px458 plasmid fades away gradually (36), the population of genome-edited cells in the cell cultures can be monitored by the presence of fluorescence (*SI Appendix*, Fig. S2 *A* and *D*). In all cases presented here, most cells that survived after puromycin treatment readily showed a green fluorescent signal putatively originating from inserted mNeonGreen (Fig. 2*A*), thus no further enrichment via FACS was performed. Three forms of HDR donor all resulted in efficient tagging, and western blotting confirmed the molecular weight of tagged protein including the introduced purification tags (Fig. 2 *A* and *B*). Note that this theoretical molecular weight does not include puromycin N-acetyltransferase and mNeonGreen, indicating that efficient posttranslational cleavage at P2A sites. Thus, the endogenous protein is minimally perturbed with only a small affinity tag at its terminus.

In Jurkat cells, which are commonly used in studies of acute T cell leukemia, T cell signaling, as well as used as an expression system for various chemokine receptors (37), we transiently transfected suspension cells and successfully tagged five out of six targeted proteins except for VIM (Fig. 2 *C* and *D*, and *SI Appendix*, Fig. S2 *E* and *G*). Among them, the endogenous expression level of FASN in Jurkat cell is much lower than in HEK293T cells, demonstrating that tagging with our approach is not dependent on a high expression level of the target protein. Because the plasmid HDR donor produced satisfactory results, we did not test dsDNA and ssDNA HDR donors. As for HEK293T cells, the majority of Jurkat cells surviving after puromycin treatment displayed fluorescence signal (Fig. 2*C*). Western blots with an anti-FLAG antibody confirmed success in tagging the target proteins (*SI Appendix*, Fig. S2*E*). The morphology and average size of genome-edited Jurkat cells remained comparable to the control, suggesting that major cellular processes, such as metabolism, signaling cascades, and environmental response, remain normal (*SI Appendix*, Fig. S2 *F* and *G*). Intensity of the selection marker mNeonGreen correlates roughly to the cellular mRNA level in Jurkat cells (34, 35) (*SI Appendix*, Fig. S2*G*). Measuring the fraction of cells that display green fluorescence signal allows us to estimate the knock-in efficiency (Fig. 2*D*). Clearly, there are still remaining populations of nonedited cells in TKT and PCNA, in which cases, further FACS sorting would be beneficial.

As a proof of principle, using only plasmid HDR template, we also tagged two proteins, GAPDH and FASN, in a breast cancer cell line MDA-MB468 with a FLAG tag. Both fluorescent signal and anti-FLAG western blot confirm the success of tagging (*SI Appendix*, Fig. S2 *H* and *J*). In both cases, with only puromycin selection, the fractions of genome-edited cells are lower than that of tagging the same targets in the Jurkat cell (*SI Appendix*, Fig. S2*I*). Further FACS sorting may help to increase the population of genome-edited cells.

### Characterization of Endogenously Tagged Proteins.

**Fig. 3.**
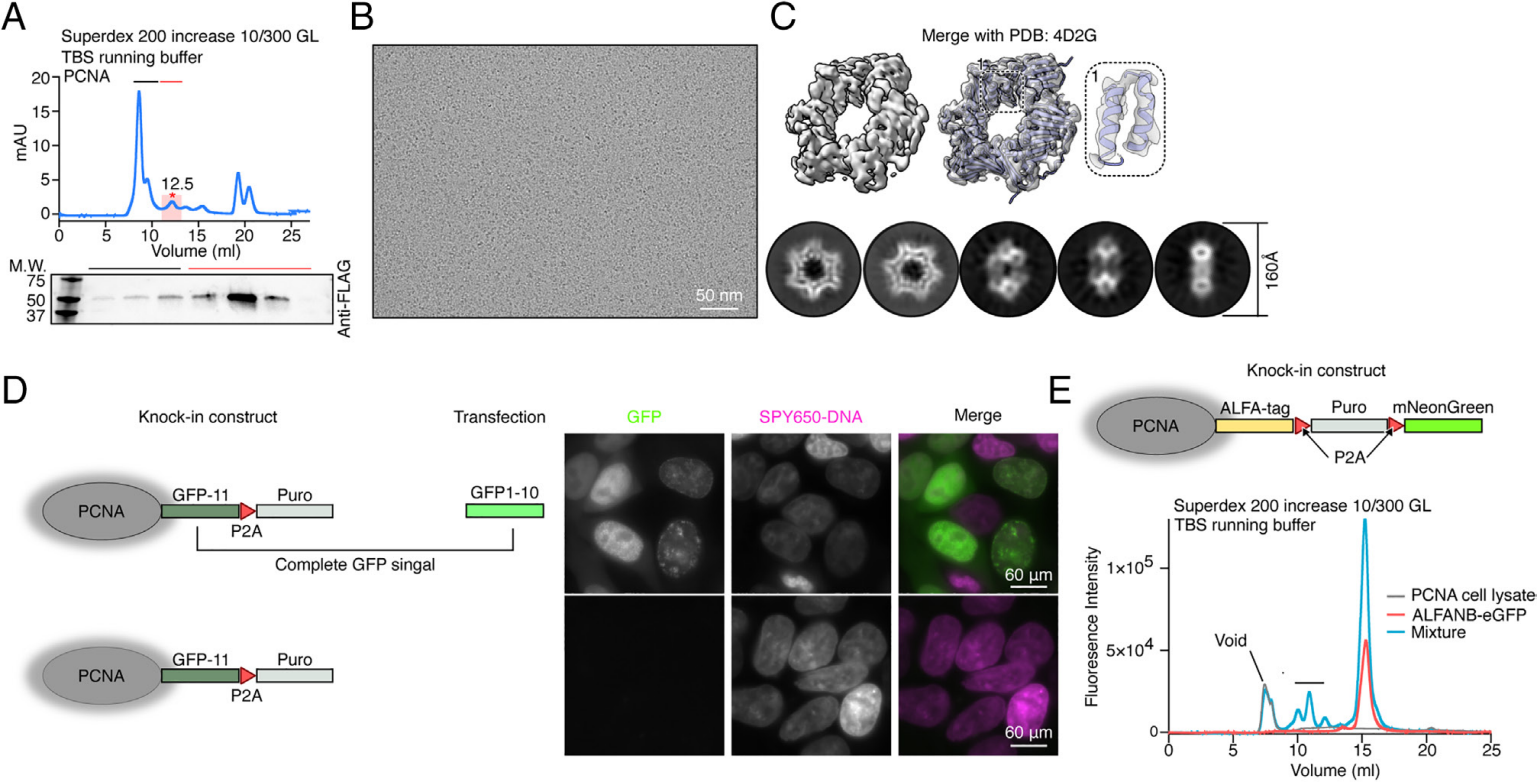
Characterization of endogenous PCNA. (*A*) SEC profile of 3×FLAG-tagged endogenous PCNA after affinity purification by anti-FLAG M2 resin. Colored labels (black and red line) above the SEC profile match with the corresponding fractions in the western blot. The pink box on the profile indicates the theoretical elution volume of PCNA. (*B*) Cryo-EM micrograph of purified endogenous PCNA. (Scale bar: 50 nm.) (*C*) 3D reconstruction of endogenous trimeric PCNA. *Left*: sharpened density map. *Right*: density map with the atomic model (PDB: 4D2G) docked. The small insert shows that helices are resolved. The bottom row shows selected 2D class averages of PCNA particles. (*D*) With the affinity purification tag being replaced with GFP-11 and removal of mNeonGreen selection marker, followed by further transient transfection of genome-edited cells with GFP1-10 (*Left*, upper row), fluorescence microscopy shows that PCNA is located in nucleus (*Right*, upper row). As a control, without transient transfect genome-edited cells with GFP1-10 (*Left*, bottom row), no fluorescence signal was detected. The nucleus is visualized by staining DNA with SPY650-DNA. (*E*) In combination with ALFA-tag and GFP-conjugated ALFA nanobody (ALFANB-eGFP), we enabled FSEC of PCNA (blue profile) directly from cell lysate. As controls, FSEC profiles of cell lysate without ALFANB-eGFP (gray profile) shows a flat profile. FSEC of purified ALFANB-eGFP alone (red profile) indicates the position of the ALFANB-eGFP alone. Together, the peaks in the blue profile marked by a black line are a putative complex of PCNA. The experiment was repeated three times.

With the targeted proteins tagged with an affinity tag, we proceed to purify them from HEK293T cells for further characterization. To produce enough cells for protein purification, we transferred and expanded edited cells in suspension. For all six targets, we purified proteins by affinity pulldown followed by size exclusion chromatography (SEC), validation by anti-FLAG western blot, and visualization by negative stain EM (Fig. 3 *A* and *B* and *SI Appendix*, Fig. S3).

We chose PCNA as an example, which is essential for DNA replication and mostly localized within the nucleus and forms different complexes with multiple interaction partners (38–40), to demonstrate our applications. We tagged PCNA with a 3×FLAG tag and purified endogenous trimeric PCNA by affinity purification followed by SEC and validation by anti-FLAG western blot (Fig. 3*A*). The negative stain EM shows a rather heterogenous distribution of particles, but a large portion of particles have the size and shape of trimeric PCNA (Fig. 3*B*). Without further purification, we subjected the sample to single-particle cryo-EM analysis using functionalized graphene oxide (GO) affinity grids (41) and determined a structure of trimeric PCNA at a resolution of ~4.1 Å (Fig. 3*C* and *SI Appendix*, Fig. S4). With minor modifications of the HDR donor template, our approach can also be used to enable other applications. Adding the 11th strand of an optimized superfolder GFP (GFP-11) after the affinity tag and removing the fluorescent selection marker, followed by transient transfection of GFP1-10 into cells after puromycin treatment, we visualized localization of PCNA in the nucleus of edited cells by fluorescence microscopy (Fig. 3*D* and *SI Appendix*, Fig. S1*C*). Similarly, introducing an ALFA-tag to PCNA and incubating eGFP conjugated ALFA-nanobody (42) with cell lysate enabled FSEC profiling of PCNA (Fig. 3*E* and *SI Appendix*, Fig. S1*D*). Broadly, FSEC profiling enables efficient optimization of endogenous protein purification and high-throughput screening of small molecules to disrupt or enhance endogenous assembly of functional complexes.

In addition to PNCA, we also determined structures of endogenous human FASN (6.8 Å) and GAPDH (3.9 Å), all by using a 200 kV electron microscope (*SI Appendix*, Fig. S4). We also determined cryo-EM reconstructions of two co-purified proteins, 20S proteasome at a low resolution due to a limited number of particles isolated from a dataset collected from purified GAPDH sample and methylosome at 2.9 Å resolution from sample collected from a SEC peak of FASN (*SI Appendix*, Fig. S4).

### Characterization of Endogenous GAPDH.

To demonstrate that characterization of endogenous proteins can provide structural and functional insights not observed in previous studies using recombinant protein, we focused on endogenous GAPDH. GAPDH is a critical enzyme in the glycolysis pathway, and it mainly localizes in the cytoplasm. Malfunction of GAPDH lowers cellular energy generation and triggers cell death (43, 44). Furthermore, GAPDH also promotes phosphatase activity against both PtdIns (3,4,5)P_3_ and PtdIns (4,5)P_2_ (45). Structurally, the catalytic reaction process of GAPDH is well studied from recombinant proteins (46, 47).

**Fig. 4.**
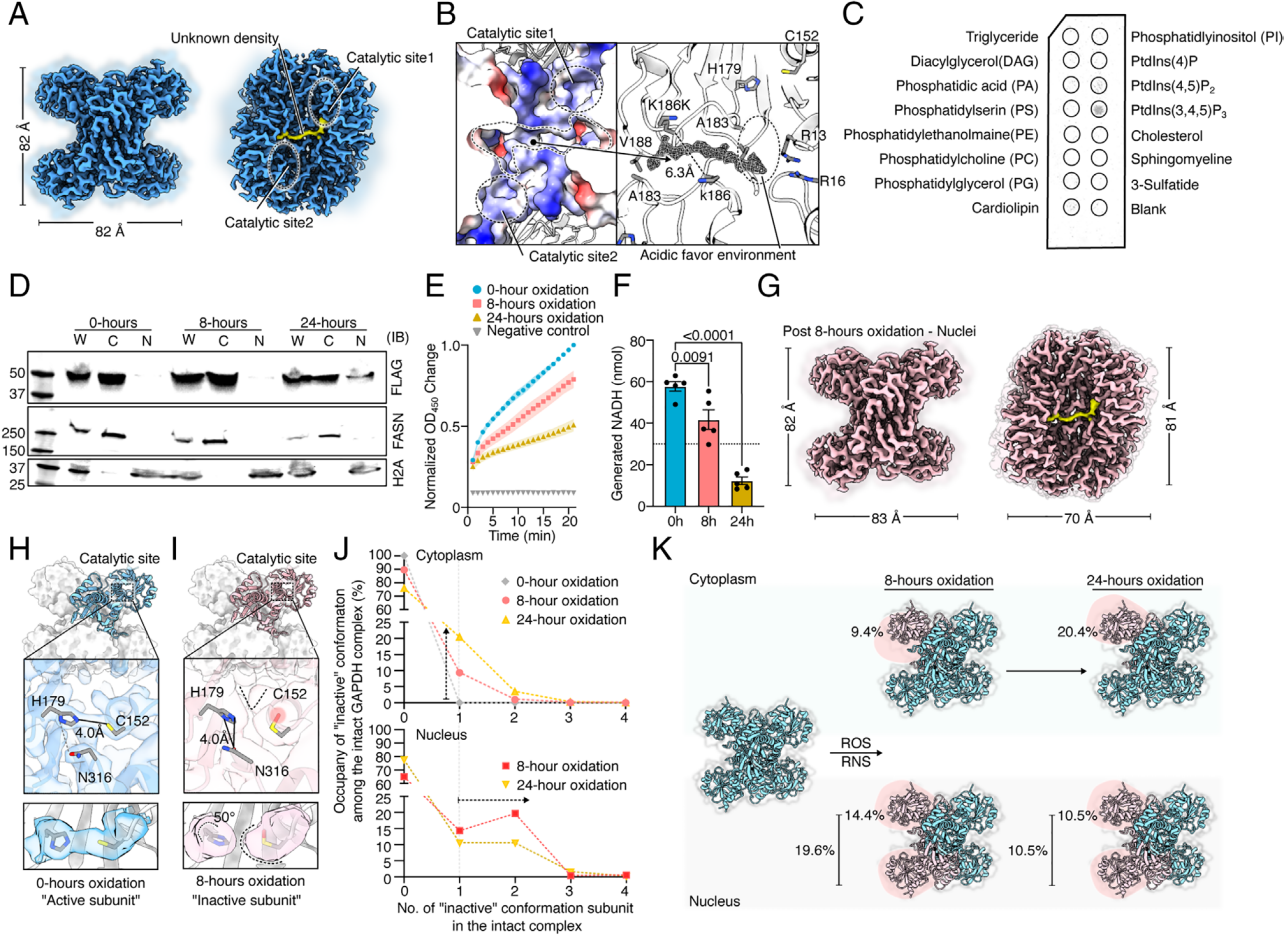
Structural changes of human endogenous GAPDH in response to prolonged oxidative stress. (*A*) Two different views of the human endogenous GAPDH cryo-EM map determined without oxidative stress. A nonprotein density (yellow) is seen connecting the catalytic site (circled with a dashed line) from two neighboring subunits. (*B*) An enlarged view of the nonprotein density. *Left*: The electrostatics surface of the groove surrounding the nonprotein density. The locations of the nonprotein density and the catalytic sites nearby are marked by dashed lines. *Right*: Nonprotein density is shown in mesh. The groove is formed mainly by backbones of the same loop from two neighboring subunits. R13 and R16 provide positively charged local environment for substrate recruitment. (*C*) The lipid strip assay shows delipidated endogenous GAPDH preferentially binds to PIP_3_. The experiment is repeated twice independently. (*D*) Western blots of FLAG-tagged endogenous GAPDH (top row) from lysate of whole cell (W), cytosol (C) and nuclei (N) at three time points (0, 8, and 24 h) after applying oxidative stress. As controls, western blots of FASN (middle row), and H2A (bottom row) from the same lysates show that FASN remains in cytosol and H2A in nuclei. The experiment is triplicated independently. (*E* and *F*) Upon prolonged oxidative stress, GAPDH enzymic activity decreases. The OD450 curve is normalized to the maximum point of the 0-h oxidation condition. The bar graph shows the amount of generated NADH, and the statistical significance is tested by the two-tailed paired *t* test. The *P*-values are shown on the graph. The experiment is repeated multiple times (in *SI Appendix*, Fig. S6). (*G*) Cryo-EM density map of nuclear GAPDH after 8-h oxidation. (*H*) Configuration of an active catalytic triad. Without oxidative stress, the side chain of C152 and H179 is connected, with no other substrate density. (*I*) Configuration of an inactive catalytic site found from nucleus GAPDH after 8- or 24-h oxidation. C152 side chain has a bulk density, and H179 is connected with N316. (*J*) Distribution of “inactive subunits” found in the cytosolic (*Upper*) and nucleus (*Bottom*) GAPDH complex. Intact GAPDH particles with two inactive subunits are mainly found in nuclei. (*K*) Illustration of the impact of prolonged oxidative stress [reactive oxygen species (ROS) and reactive nitrogen species (RNS)] on the number of inactive subunits in GAPDH particles found in cytosol and nuclei.

By collecting a larger dataset with a 300-kV electron microscope, we determined a high-resolution structure (2.0 Å resolution) of human endogenous GAPDH from HEK293T cells (Fig. 4*A* and *SI Appendix*, Figs. S5 and S6), in which we observed a continuous nonprotein density nested within a groove formed in between two monomers and connecting the catalytic sites of two neighboring monomers. The surface of this groove is relatively hydrophobic, except for a pair of lysine residues (K186 from two opposing monomers) with their charged head groups pointing away from the groove toward solvent (Fig. 4*B* and *SI Appendix*, Fig. S7*A*). Considering that its shape resembles a single fatty acid chain nested within a hydrophobic groove and that GAPDH can attach to membranes and stimulate phosphatase activity against PIP_3_ and PIP_2_ (45, 48, 49), we modeled this density as a lipid. To support this speculation, we used detergent to remove any bound lipids from purified GAPDH and applied this lipid-free GAPDH to a premade lipid strip. Subsequent western blotting showed that PIP_3_ indeed binds to GAPDH (Fig. 4*C* and *SI Appendix*, Fig. S7*B*). To further test the functional significance of binding PIP_3_ in this groove, we introduced point mutations (K186E, A183W, V188W, R13E, and R16E, and a double mutation of A183W/V188W) to recombinant GAPDH overexpressed in Expi293, because it is often easier to assay the effects of mutations with recombinant proteins than with endogenous proteins. All these point mutations were designed to disrupt the interactions between GAPDH and the bound PIP_3_. Among them, R13 and R16 are in the groove where the negatively charged headgroup of the alleged PIP_3_ may bind (Fig. 4*B* and *SI Appendix*, Fig. S7*A*). R13 is highly conserved and is known to be required for nicotinamide adenine dinucleotide (NAD^+^) recruitment (50, 51). Indeed, recombinant proteins with these mutations, except the one with single A183W mutation, are found largely in monomer form (*SI Appendix*, Fig. S7 *C* and *D*). We thus speculate that PIP_3_ could be a natural resource to stabilize the oligomerization interface acting as hydrophobic glue and providing a phosphate source for catalytic reactions. Competitive binding of small molecule drugs can potentially disrupt cellular GAPDH (46).

### GAPDH in Response to Oxidative Stress.

GAPDH function is highly sensitive to the redox environment within the cell (52). Prolonged oxidative stress oxidizes GAPDH, which is subsequently translocated into the nucleus and initiates apoptosis (53, 54). We applied oxidative stress to genome-edited HEK293T cells and purified endogenous GAPDH from cytosol and nucleus separately after 8 and 24 h of oxidative stress (Fig. 4*D*). Indeed, prolonged oxidative stress leads to translocation of GAPDH from cytosol to nucleus (Fig. 4*D*) and is accompanied by a decrease of GAPDH activity (Fig. 4 *E* and *F*, *SI Appendix*, Fig. S8). We further determined structures of cytosolic and nuclear GAPDH from oxidatively stressed cells (Fig. 4*G* and *SI Appendix*, Figs. S5 and S6). Interestingly, by analyzing structure of individual subunits from all GAPDH datasets, including the one without oxidative stress, we captured different conformations of the GAPDH catalytic site (*SI Appendix*, Fig. S9). In one conformation, there is a density connecting side chains of C152 and H179 (Fig. 4*H* and *SI Appendix*, Fig. S9). Considering that this is the only conformation of the catalytic site we found in GAPDH without oxidative stress, we name it as the active subunit (Fig. 4*H* and *SI Appendix*, Fig. S9). In another conformation, which we only found in oxidatively damaged GAPDH, H179 is no longer connected to C152 but instead connected to N316 through a weak density and C152 has a bulky side chain density, as observed in oxidized cysteine found in other studies (55) (Fig. 4*I* and *SI Appendix*, Fig. S9). Thus, this conformation likely represents the oxidatively damaged catalytic triad, in which oxidized C152 is no longer hydrogen bond with H179. We name this conformation as inactive subunit.

By counting the numbers of two types of subunits in all structures, we observed an increase of catalytically inactive subunits in both cytoplasmic and nuclear GAPDH upon prolonged oxidative stress (Fig. 4*J*). In the cytoplasm, the fraction of GAPDH containing one inactive subunit increases from zero to ~10% after 8 h and ~20% after 24 h (Fig. 4*J* and *SI Appendix*, Fig. S9*B*). In the nucleus, we obtained structures of GAPDH only after 8 and 24 h oxidative stress. The number of particles containing one inactive subunit remains at ~15%, but the number of particles containing two inactive subunits is between 10% and 25% (Fig. 4*J* and *SI Appendix*, Fig. S9*B*). Note that we did not account for the subunit with its conformation of the catalytic site not clearly defined. Nonetheless, our data reveal a trend that the increased number of inactive subunits plays a role in translocating GAPDH from the cytoplasm to the nucleus (Fig. 4 *J* and *K*).

## Discussion

Proteins are dynamic macromolecules that can be regulated, modified, compartmentalized, and more, all depending on their cellular environment. With recent advancements in structural biology, particularly the technological breakthroughs in single-particle cryo-EM and artificial intelligence driven protein structure prediction (56, 57), structural biology is now extended beyond determining atomic structures toward characterizing how proteins function in their complex native environment. The ability to tag endogenous proteins in mammalian cells enables structural studies of challenging targets in their native environment, in carrying out biological processes and in response to external stimuli. Combining it with mass spectrometry would also enable characterization of the interactome in mammalian cells. In this study, we demonstrate the versatility of our HDR donor template approach in efficient CRISPR/Cas9 tagging of six endogenous proteins in two different mammalian cell lines, HEK and Jurkat, and two of them in a breast cancer cell line, MDA-MB468. Taking GAPDH as an example, we demonstrate capturing its spatial and temporal structural changes within cells in response to oxidative stress.

Each genome-editing tool has its specific purpose and criteria of success. To characterize endogenous proteins biochemically and biophysically, it is necessary to ensure that the majority of cells in the final culture are genome-edited for efficient targeted protein purification. This requirement imposes challenges on how to efficiently isolate genome-edited cells from the initial culture such that the final culture contains mostly the genome-edited cells. The most commonly used schemes include chemical selection, which was demonstrated previously (58–61), or FACS, which was used in our previous study (26). The advantages and limitations of these selection schemes individually or in combination are reviewed recently (62). Our double selection marker approach combines the two, in which we use a continuous or interval antibiotic treatment to eradicate unedited cells progressively and use a florescence marker to monitor the enrichment of genome-edited cells in the culture, followed by final florescence-based selection if necessary (Fig. 2 *B* and *G*). Together with the accumulation of the FP separated from the target, it facilitates a more efficient FACS sorting of genome-edited cells if such further enrichment is necessary. A similar double selection scheme was also used previously by others for different purposes (63, 64).

Another technological advantage of our design is that three types of commonly used HDR donor (ssDNA, dsDNA, and plasmid) can be generated from the same plasmid. These different forms of HDR donors can be tested in parallel, or even together in the same target cells, thus increasing the success rate.

A brief comparison of our approach with the methods described in previous studies is illustrated in *SI Appendix*, Fig. S10. Together, our approach makes the final success rate of tagging endogenous proteins less dependent on the initial efficiency of knock-in. Thus, instead of using viral infection, our procedure is entirely carried out by transient transfection. This is practical beneficial when editing some cell lines, such as Jurkat cells that are considered to be hard to manipulate. In this study, in less than 2 weeks, we tagged six genes in HEK293T and Jurkat cells. However, we see no obvious obstacle in tagging other proteins in these cell lines or extending this strategy to other cell types. As examples, we tagged GAPDH and FASN in the breast cancer cell line MDA-MB468 (*SI Appendix*, Fig. S2 *H* and *J*), although no further analysis was performed at this stage.

There are also limitations, such as that the tagging strategy presented here is restricted to the N or C terminus of target proteins and the results of tagging are often heterozygous, although termini are the most common tagging sites for structural biology and heterozygous tagging does not impair protein purification. For endogenous proteins with very low expression levels, antibiotic selection can be toxic even to successfully genome-edited cells. In such cases, an optimized antibiotic selection protocol, with reduced antibiotic dosage and intermittent antibiotic addition intervals, may have to be applied.

While there are many advantages in studying endogenous proteins, it is not a replacement of recombinant technology. For example, classic approaches of dissecting protein functions by introducing point mutations, domain deletions or swapping, etc., can easily be carried out with recombinant proteins but may not be achievable using endogenous proteins. As demonstrated with GAPDH in this study, introducing point mutations is an effective way to test the significance of the lipid binding in terms of GAPDH function. The outcomes of designed point mutations can be easily assayed with recombinant proteins but difficult with endogenous proteins if such mutations interfere with protein function and lead to cell death.

In summary, our approach, when combined with other biophysical methods like mass spectrometry, single-particle cryo-EM, and tomography, enables studies of native protein behaviors under different intrinsic and extrinsic stimuli that cannot be captured with standard heterologous expression systems.

## Materials and Methods

### Plasmid YifanCheng (pYC) Generation, Homology Arm Design, and Single-Guide RNA (sgRNA) Generation.

The backbone of pYC was derived from a pEG plasmid with plasmid amplification and antibiotic resistance. The tag components, purification tags, and selection markers, were synthesized by Integrated DNA Technology and subcloned by PCR. The choices of affinity tag, antibiotic resistance gene, self-cleavage peptide, FPs, or their combinations for different applications are summarized in *SI Appendix*, Fig. S1. The generated pYC were then amplified using the DH5α competent *E. coli* cells. The designed multiple cloning sites were validated by double-restriction digestion and subsequent agarose gel analysis. The pYC sequences were confirmed via Sanger sequencing (Elim BioPharma).

The left and right HDR arms were designed based on the CRch38 (hg38, *Homo sapiens*) in Benchling (https://benchling.com/) and NC_000012.12 Chromosome 12 Reference GRCh38.p14 Primary Assembly in NCBI database (https://www.ncbi.nlm.nih.gov/gene). To achieve optimal knock-in and cost-efficiency, the length of the arms was maintained within a range of 500 to 990 bp, including sticky and blunt enzyme digestion sites. Both arms were subcloned into the pYC plasmid by PCR or by the digestion-ligation method. To generate the double-stranded DNA form of the HDR, specific blunt cutter sites on MCSs were introduced by PCR and the reconstituted plasmid was digested at 37 °C for 2 h by the corresponding enzymes, followed by agarose gel purification (Qiagen). To obtain the single-stranded DNA form of the HDR, the HDR was first amplified by PCR using a pair of primers (Elim BioPharma) in which the 5′ end of the 3′ primers is modified by addition of a phosphoryl group. The PCR product was then incubated with 1 uL of lambda exonuclease (NEB), which recognizes this phosphoryl group on the 5′ end of DNA and degrades one strand to leave behind ssDNA, at 37 °C for 0.5 to 1 h. Due to the lack of DNA secondary structure, SYBR Gold (ThermoFisher) was applied for the visualization. The digested samples were then purified by the DNA purification kit (Zymo research) and diluted into the designated concentration.

The sgRNAs were designed using CRch38 (hg38, *Homo sapiens*) in Benchling software. The highest-ranked sgRNA fragments were synthesized by Elim Biopharm and subcloned into the plasmid px458 as previously described (65). In brief, a pair of primers, which encodes sgRNA and a BbsI site (NEB), were synthesized (Elim BioPharma), and two pairs of primers were annealed in a thermocycler. The annealing product was ligated into prelinearized px458 using T4 ligase (NEB).

All DNA materials were stored at −20 °C until their usage.

### Stable Cell Line Generation.

Adherent HEK293T cells were grown in DMEM (Gibco) containing 10% fetal bovine serum (FBS) (v/v) at 37 °C and 8% CO_2_. Upon reaching 50% confluence at one well in a 6-well plate, cells were transfected with 300 ng of px458, or px458-mApple, and 500 ng of HDR templates (be it plasmids, dsDNA, or ssDNA) using Lipofectamine 2000 (ThermoFisher). After the cell number is quadrupled and typically after 2 d, cells were treated continuously with puromycin (ThermoFisher) at a concentration of 1 to 2 μg/mL in culture. Continuous puromycin treatment eradicates unedited cells during 10 to 14 d (10 to 14 cycles of selection), followed by confirming successful knock-in with fluorescence microscopy, western blot, and cDNA sequencing. After confirmation, the cells were transferred to T75 flask and grown to approximately 70% to 90% confluence. Then, the cells were suspended by using 20 mL of suspension culture media, Freestyle293 media (Gibco) with 1% FBS (v/v). This normally takes a week. The suspended cells were grown in 37 °C with 5% CO_2_ by shaking at 120 rpm. Again, puromycin was added to 100 mL of suspension culture at a concentration of 1 μg/mL, followed by growing cells further without supplement of additional puromycin until cell culture is expanded to 400 to 800 mL for protein purification. In summary, 400 to 800 mL cultures of cells sufficient for protein purification were obtained within a month posttransfection.

Jurkat cells were grown in home-modified RPMI 1640 media (Gibco) supplemented with 10% FBS (v/v), 100 U/mL of penicillin-streptomycin, 2 mM of L-glutamine, 10 mM HEPES pH 7.4, and 1 mM sodium pyruvate. At a cell density of 1 to 1.5 × 10^6^ cells/mL, knock-in reagents (300 ng of px458 and 500 ng of HDR template) were delivered to the cells using Lipofectamine 2000 (ThermoFisher). After 3 d, 1 μg/mL of puromycin was added for knock-in selection, typically for 10 to 14 d (10 to 14 cycles of selection). Dead cells were removed by centrifugation at 100*g* for 3 min at room temperature.

MDA-MB468 cells were grown in home-made media (50% (v/v) fresh DMEM combined with 50% (v/v) of conditioned media) supplemented with 10% FBS (v/v). When the cell density reached 70% confluency of a well in a 6-well plate, the knock-in materials (500 ng of px458 and 1,500 ng of HDR template) were delivered to the cells using Lipofectamine 3000 (ThermoFisher). After 3 d, 1 μg/mL of puromycin was added for selection of edited cells in an interval manner for approximately 30 d (8 to 10 cycles of selection).

To store genome-edited cells, HEK293T and Jurkat cells were resuspended with corresponding fresh media without puromycin and spun down at 1,000*g* for 5 min and resuspended at a density of 1 × 10^7^ cells/mL per vial. Cell pellets were resuspended with freezing media [50% fresh media, 40% conditioned media and 10% DMSO (Sigma)] and transferred to cryogenic vials (Corning). The vials were slowly frozen in Mr. Frosty at −80 °C for 8 to 12 h and transferred to liquid nitrogen dewar for long-term storage.

### Knock-in Validation.

For western blot analysis, cell pellets were resuspended in 180 μL of ice-cold TBS buffer (20 mM Tris-HCl, pH8.0, 150 mM NaCl) and then combined with 20 μL of 100 mM n-dodecyl-β-D-maltoside (DDM) and 20 mM cholesteryl hemisuccinate (CHS) to lyse cells. The mixtures were rotated at 4 °C for 45 min before 4xSDS-loading buffer was added (Bio-Rad). The samples were vortexed, boiled at 90 °C, spun down at 20,000*g* for 1 min at 4 °C in a bench-top centrifuge (Eppendorf 5810R) and subjected to SDS-PAGE gel (Bio-Rad, 4 to 15% gradient). Proteins were then transferred to a 0.2 μm nitrocellulose membrane (Bio-Rad) using a Trans-Blot Turbo (Bio-Rad). The samples were immunoblotted using anti-FLAG-peroxidase (HRP) (Sigma-Aldrich, A8592) to validate their sizes. The peroxidase signals were developed with SuperSignal (ThermoFisher) and imaged in a ChemiDoc MP (Bio-Rad).

For fluorescence microscopy, the cells were seeded into 8-well chambered #1.5 coverglasses (C8-1.5P, Cellvis) coated with poly-L-lysine (Sigma-Aldrich) or fibronectin (Corning). Samples were imaged in DMEM FluoroBrite (Gibco) supplemented with 10 % FBS (UCSF Cell culture facility) and SPY650-DNA (Cytoskeleton Inc.) Widefield fluorescence microscopy was performed on a custom-built microscope constructed around a Ti-E body (Nikon) equipped with a water immersion objective (CFI Plan Apochromat IR 60x WI NA 1.27, Nikon), an active focus stabilization system (PFS, Nikon) and a motorized stage (MS-2000, ASI). A LED light source (X-Cite XLED1, Excelitas) operated at excitation power densities of 0.7 W/cm^2^ at 488 nm and 1.6 W/cm^2^ at 640 nm at the sample plane was used for excitation of mNeonGreen and SPY650-DNA. An sCMOS camera (Orca Flash 4.0, Hamamatsu) with a back-projected pixel size of 108 nm was used to detect bright-field and fluorescence signals. The sample was maintained at 37 °C and 5% CO_2_ using a stage-top incubation chamber with environmental control unit (Tokai Hit). Emitted fluorescence was separated from excitation light using a 405/488/561/640 nm quadband dichroic and mNeonGreen and SPY650-DNA signal was further filtered using 525/50- and 700/75-nm bandpass filters. All microscope components were controlled using the micromanager 1.4 software platform (66).

Total RNA was extracted using a Monarch total RNA miniprep kit (NEB) following the protocol provided by the manufacturer. The concentration of extracted total RNA was measured with a NP80 Nanophotometer (Implen) and 1 μg of total RNA was subjected to reverse transcription reaction to generate cDNA using LunaScript RT super mix (NEB). For the PCR reaction, a gene specific 5′ primer and common 3′ primer (3xFLAG) were used as listed below. The amplicons were isolated from a 1% agarose gel using PCR purification kits (Acros and Qiagen). Purified amplicons were sequenced by Elim BioPharma. From HEK293T cells, the full-length cDNA of *ACTB*, *GAPDH*, *TKT*, *VIM*, and *PCNA* were validated. The cDNA of *ACTB* and *PCNA* from the Jurkat cells were achieved. Extraction of full-length cDNA of *FASN* from HEK293T knock-in cells and *FASN*, *GAPDH* and *TKT* from knock-in Jurkat cells were unsuccessful. Instead, other biochemical validations, including western blot and mass spectrometry along with structure, validated successful tagging.

*ACTB*: ATGGATGATGATATCGCCGCGCTCGTCGTCGA;

*FASN*: ATGGAGGAGGTGGTGATTGCCGGCATGTCCGG;

*GAPDH*: ATGGGGAAGGTGAAGGTCGGAGTCAACGG;

*TKT*: ATGGAGAGCTACCACAAGCCTGACCAGCAGAAGCTG;

*VIM*: ATGTCCACCAGGTCCGTGTCCTCGTCCTCCTA;

*PCNA*: ATGTTCGAGGCGCGCCTGGTCCAGGGCTCC;

3xFLAG: GAGCCGAACCGTTGGTTTATACTCTGTCAT

### Endogenous Protein Purification and Subcellular Fractionation.

We only purified tagged endogenous proteins from HEK293T cells. The knocked-in cells were grown to a density of 3 to 4 × 10^6^ cells/mL in 400 to 800 mL cultures before being harvested by centrifugation at 3,000 to 4,000*g* for 10 min at 4 °C in a bench-top centrifuge (Eppendorf 5810R) or floor centrifuge (Beckman Coulter rotor JLA-8.1). The cell pellet was resuspended with ice-cold TBS buffer with additional protease inhibitor cocktail (Sigma-Aldrich). Sonication was applied to break cell membranes on ice–water mixture with gentle stirring to prevent the samples from overheating. The cell debris was then discarded by two follow-up centrifugation steps, one at 8,000*g* for 20 min at 4 °C using the rotor JA-25.50 (Beckman Coulter), and then at 126,000*g* for 1 h at 4 °C using the rotor Ti45 or 50.2Ti (Beckman Coulter), sequentially. The final supernatant was applied to pre-equilibrated 1 mL of anti-FLAG M2 affinity gel (Sigma-Aldrich) and incubated between 2 h and overnight. The beads were then loaded in a polyprep column (Bio-Read) and then extensively washed with 50 column volumes of 500 mM NaCl and 20 mM Tris-HCl pH 8.0, followed by 50 column volumes of TBS buffer. To elute the proteins, 2 column volumes of TBS buffer supplemented with 0.25 mg/mL of 3×FLAG peptide (Sigma-Aldrich) was applied twice. The eluted proteins were further purified by loading onto the size-exclusion chromatography column in TBS buffer, Superose 6 increase 10/300 GL or Superdex 200 increase 10/300 GL (Cytiva) depending on the size of the proteins and putative complexes of interest. The fractions containing the target proteins were determined by anti-FLAG immunoblotting.

To purify endogenous GAPDH from cytosol and nuclei separately, pelleted cells were resuspended with ice-cold buffer of 250 mM sucrose, 5 mM MgCl_2_, and 10 mM HEPES pH 7.4 (Fractionation Buffer) supplemented with protease inhibitor cocktail. The cells were mechanically homogenized using 10 to 20 times up-and-down in tissue glider (Wheaton). The homogenized samples were then spun down at 600*g* for 10 min at 4 °C in a bench-top centrifuge (Eppendorf 5810R). The pellet mainly contains larger components, like unbroken cells and nuclei, while the supernatant contains mostly cytoplasmic components, including lighter cellular compartments, such as the endoplasmic reticulum. Supernatants were collected for purification of cytosolic GAPDH following the procedure described above. Pellets were suspended in ice cold TBS buffer followed by sonication to break the nuclear membrane. Nuclear GAPDH was then purified following the similar procedure described above. Western blots against H2A and FASN (Fig. 4*D*) confirm efficient separation of nuclei and cytoplasm.

### Recombinant GAPDH with Point Mutations.

Human GAPDH (Uniprot ID: P04406) was subcloned into pEG plasmid containing C-terminal eGFP (Addgene ID: 160681). All mutations were generated by the overlapping PCR method and subcloned into the pEG C-terminal eGFP vector. The sequences were confirmed by Sanger sequencing (Elim BioPharma). After validating the sequence, the plasmids were transfected into HEK293T cells by using Lipofectamine 2000 and placed them at 37 °C with 8% CO_2_. Twenty hours posttransfection, 10 mM sodium butylate was added and the cells were transferred to 30 °C with 5% CO_2_. After 20 to 24 h, the cell pellets were harvested and resuspended in 180 μL of ice-cold TBS buffer and then combined with 20 μL of 100 mM DDM and 20 mM CHS to lyse cells**.** The mixtures were rotated at 4 °C for 45 min followed by spin down at 21,000*g* for 20 min at 4 °C. The sample was injected onto a Superdex 200 increase 10/300 GL column (Cytiva), pre-equilibrated with TBS buffer, at a flow rate of 0.5 mL/min. The GFP signal was collected by HPLC (Shimazu) equipped with the RF-20A fluorescence detector (excitation 488 nm and emission 508 nm, Shimazu).

### Fluorescence Size-Exclusion Chromatography (FSEC) and Fluorescence Light Microscopy.

Fluorescence size exclusion chromatography (FSEC) is an efficient method to efficiently characterize protein behavior without purification (67). FSEC is typically performed on proteins tagged with GFP. We demonstrated using FSEC to characterize PCNA without tagging it with GFP. Cell pellets were harvested from genome-edited cells and resuspended in 180 μL ice-cold TBS buffer and then combined with 20 μL of 100 mM DDM and 20 mM CHS to lyse cells**.** The mixtures were rotated at 4 °C for 45 min followed by spin down at 21,000*g* for 20 mins at 4 °C. The supernatant was incubated with 1 μL of ALFANB-eGFP, 0.1 mg/mL stock, for 2 h at 4 °C, followed by injecting onto a Superdex 200 increase 10/300 GL column (Cytiva), pre-equilibrated with TBS buffer, at a flow rate of 0.5 mL/min. The GFP signal was collected by HPLC (Shimazu) equipped with an RF-20A fluorescence detector (excitation 488 nm and emission 508 nm, Shimazu).

To visualize cellular location of PCNA-GFP-11, 30 to 50 ng of Superfolder GFP1-10 in pEG plasmid was transfected into the PCNA knock-in cells without mNeonGreen. The cells were grown in 37 °C, 8% CO_2_ for 20 h before imaging. Fluorescence imaging was performed as described above.

### Endogenous GAPDH Translocation and Enzymatic Activity.

When the GAPDH knock-in cells reached a density of 2.8 to 3 ×10^6^ cells/mL, the cells were subjected to oxidative stress by addition of 1 mM H_2_O_2_ and 10 mM L-Arg at pH 7.4 to the media at 37 °C with 5% CO_2_ by shaking at 120 rpm. After 8 and 24 h of oxidative stress, 50 mL of cells was harvested. The cells were spun down at 1,000*g* for 10 min at 4 °C using a bench-top centrifuge (Eppendorf 5810R), the supernatant was decanted, and the pellet flash-frozen in liquid nitrogen before −80 °C storage for later use.

The thawed pellet was resuspended in 10 mL of ice-cold Fractionation Buffer by gentle pipetting. Then subcellular fractionation was performed as described above. No further manipulation was done to the cytoplasmic fraction, but the nuclear fraction was rinsed in ice-cold TBS buffer twice before solubilization with 10 mM DDM and 2 mM CHS for an hour on a rotator at 4 °C. Each sample was loaded with SDS-loading buffer containing β-mercaptoethanol and boiled at 90 °C. The boiled samples were spun down and loaded onto a SDS-PAGE gel (Bio-Rad, 4 to 15% gradient). The proteins were transferred to a 0.2 μm nitrocellulose membrane (Bio-Rad) using a Trans-Blot Turbo (Bio-Rad). The samples were immunoblotted using the corresponding antibodies: anti-FLAG-peroxidase (HRP) (Sigma-Aldrich, A8592) to label the GAPDH, anti-FASN-HRP (Abcam, EPR7466) to monitor the cytoplasmic fraction, as well as anti-H2A (BioVision, cat3621-100) with its secondary antibody rabbit-HRP (Bio-Rad, cat170-6516) to confirm the nuclear fraction. The peroxidase signals were developed with SuperSignal (ThermoFisher) and imaged by ChemiDoc MP (Bio-Rad).

GAPDH in the cytosolic compartment was also assessed for its activity. GAPDH levels were estimated by immunoblotting to keep amounts constant during the assay. The same quantities of proteins from the different oxidation stress conditions (none, 8 h, and 24 h) were added onto Greiner 96-well flat-bottom transparent plates, and GAPDH enzymic activity was assessed with a GAPDH activity assay kit (Abcam, ab204732). Endogenous GAPDH catalyzes conversion of glyceraladehyde-3-phophate into 1,3-bisphosphate glycerate while converting nicotinamide adenine dinucleotide (NAD^+^) to NADH. Product formation was measured in a colorimetric assay by absorption at 450 nm on a SPARK 10 M plate reader (TECAN). Samples were incubated at 37 °C and agitated every 5 s, while OD450 was measured every minute for 30 min.

### Delipidation of Endogenous GAPDH and Membrane Lipid Strip Assay.

Cells with FLAG-tagged GAPDH were grown to a density of 3 to 4 × 10^6^ cells per mL at 400 mL total culture volume. Initial purification steps were followed as described above. After sonication, the supernatant and pellet were separated through two rounds of centrifugation at 8,000*g* and 126,000*g*, respectively. The supernatant from the latter round of centrifugation was filtered through a 0.2-μm filter. For GAPDH delipidation, 10 mM lauryl maltose neopentyl glycol (LMNG) and 2 mM CHS were added to the clarified supernatant, and the mixture was incubated with 1 mL of pre-equilibrated anti-FLAG M2 affinity gel at 4 °C overnight. The beads were washed with 200 column volume of TBS buffer to remove detergent, and GAPDH was eluted with 3 column volume of TBS buffer supplemented with 0.25 mg/mL 3×FLAG peptide. The elution was further incubated with 50 mg of Bio-Beads SM2 (Bio-Rad) at 4 °C for 5 h to remove any remaining detergent micelles. The sample was then concentrated using a 50-kDa MWCO filter and ran through a Superdex 200 Increase 10/300 GL column equilibrated with TBS buffer. The delipidated GAPDH peaks were pooled and used for lipid strip assays.

The membrane lipid strip P-6002 (Echelon Bioscience) was used to identify the lipid(s) that interact with GAPDH. The membrane lipid strip was first blocked with TBST buffer [25 mM Tris-HCl, pH 7.2, 150 mM NaCl, 0.1% Tween-20 (v/v)] with 5% (w/v) milk for 1 h at room temperature and then washed three times with TBST buffer for 10 min each round. The strip was then gently agitated in 10 mL of 3 to 8 µg of purified GAPDH for 1 h at room temperature. Conventional immunoblotting was used to visualize lipid-bound endogenous GAPDH using an anti-FLAG antibody further developed with SuperSignal and imaged with ChemiDoc MP.

### Widefield Fluorescence Microscopy Data Processing.

To determine the fraction of cells exhibiting successful genomic integration, image sets of at least 20 fields of view per condition were automatically processed using custom-written analysis routines implemented in ImageJ and Matlab. First, individual cells were segmented using Cellpose (68) with custom-trained models for detection of nuclei labeled with SPY650-DNA (HEK293T, MDA-MB468 cells) or entire cells in bright-field images (Jurkat cells). From segmented regions, the mean mNeonGreen or mApple intensity as well as the segmented area was extracted for each cell. Cells were classified as expressing exhibiting a positive fluorescence signal if the mean intensity for given cells exceeded the mean + 2 SDs of the respective wild-type cell population.

### Cryo-EM Sample Preparation and Data Collection.

Purified protein samples were concentrated to 0.025 to 0.15 mg/mL using a 50-kDa cutoff protein concentrator (ThermoFisher). Then, 3 μL of the sample was applied to GO grids with amine modification [made from gold quantifoil grids, 1.2/1.3-μm size/hole space, 200 to 300 mesh by applying ethylenediamine (Sigma)] (41), blotted for 4 to 6 s with 0 blotting force at 100% humidity using a Vitrobot Mark III, and flash-frozen in vitreous liquid ethane. The grids were screened by Talos Arctica or Glacios electron microscope (ThermoFisher-FEI), operated at 200 kV, and equipped with Gatan K3 camera (Gatan, Inc.). Movies were acquired when suitable grids are identified. For the GAPDH sample, a total of five cryo-EM datasets of endogenous human GAPDH purified at different time points from cytosol and nucleus of cells after prolonged oxidative stress were collected by using the Titan Krios electron microscope operated at 300 kV and equipped with the Gatan K3 camera and BioQuantum energy filter. The energy selection slit is set to 20 eV. Movies were recorded in superresolution mode at a nominal magnification of 105 K, resulting in a superresolution pixel size of 0.4175 Å/pixel (0.835 Å/pixel after 2x FT bin). Each movie stack was dose-fractionated in 80 frames using a total exposure time of 2 s at 0.025 s per frame. The total does was 45.8 (e^−^/Å^−2^). All image stacks were collected using SerialEM (69). Defocus values varied from −0.8 to −1.5 μm.

### Cryo-EM Data Processing.

For the 200 kV datasets, all movies were motion-corrected on-the-fly by MotionCor2 (70) implemented in Scipion (71). CTF estimation and particle picking were performed using CryoSPARC (72) on motion corrected micrographs. Picked particles were subjected to 2D classification followed by discarding bad particles and pooling good ones together for further processing. Initial 3D reconstructions were generated ab initio, followed by heterogenous refinement and 3D classification to further clean up the datasets. Final datasets were subject to multirounds of iterative nonuniform refinement followed by global CTF refinement. Resolutions were estimated following gold standard by Fourier Shell Correlation (FSC) = 0.143 criterion.

For GAPDH Krios datasets, motion-correction and dose weighting were performed using MotionCor2 implemented in Relion (73). CTF was estimated by CTFFIND-4.1 (74). By using *relion_star_handler*, micrographs with lower than preset resolution were triaged. Particle picking was first performed in 50 randomly selected micrographs. A GAPDH reconstruction determined from a Glacios dataset was used as a template for particle picking. Particle picking was performed either by using Topaz (for 0-h oxidative stress) or template-based particle-picking (8-h oxidation and 24-h oxidation).

Extracted particles were first 4-binned and subjected to two rounds of 2D classification to remove junk particles. The remaining particles were re-extracted with 2-times-binning and subjected to 3D classification analysis with two different regularization value, 4 and 15 in Relion. The classification results were monitored by visualizing maps and the corresponding angular distributions in UCSF Chimera or ChimeraX (75, 76). The low-resolution GAPDH structure from Glacios dataset was used as a reference map and *C1* symmetry was applied. Several rounds of further refinements of Euler angles were performed with D2 symmetry and mask applied, followed by multiple rounds of CTF refinements, anisotropic and CTF fitting, and Bayesian polishing. Final reconstructions with D2 symmetry are between 1.97 Å to 2.33 Å. After that, for each dataset, we performed symmetry expansion from C1 reconstruction and background subtraction to isolate single subunit from the tetrameric complex, followed by 3D classification of monomer without image alignment under T = 15 and k = 5 parameter in Relion. A graphic description of the data processing workflow is illustrated in *SI Appendix*, Fig. S5. All subunits are classified into three classes, active, inactive, and not-defined (N.D.) (*SI Appendix*, Fig. S8). The assignments of inactive subunits from each tetrameric particle were counted to produce Fig. 4 *J* and *K*.

### Model Building.

Crystal structure of GAPDH (PDB:4WNC) was docked into cryo-EM density using USCF Chimera, and manually modified by using Coot (77), followed by several rounds of real-space refinement in Phenix (78). Final GAPDH models were validated in Coot and by using Molprobity (79).

## Supplementary Material

Appendix 01 (PDF)

## Data Availability

All cryo-EM density maps and coordinates are deposited to the EMDB and PDB database, with the accession number listed in *SI Appendix,* Table S1. All other materials are available upon reasonable request and completion of material transfer agreement with UCSF.
