# Supplementary material for: Efficient tagging of endogenous proteins in human cell lines for structural studies by single-particle cryo-EM
Source: Proc Natl Acad Sci U S A. 2023 Jul 24;120(31):e2302471120. doi: 10.1073/pnas.2302471120 (PMC10401002; doi:10.1073/pnas.2302471120)
Supplement: Supplementary file 1 — Appendix 01 (PDF) [file pnas.2302471120.sapp.pdf]

PNAS

Supporting Information for

Efficient tagging of endogenous proteins in human cell lines for structural studies by single particle cryo-EM

Wooyoung Choi[1†], Hao Wu[1†], Klaus Yserentant[2†], Bo Huang[2,3], and Yifan Cheng[1,4*]
[1]Department of Biochemistry and Biophysics, University of California San Francisco, San Francisco, CA 94143

[2]Department of Pharmaceutical Chemistry, University of California San Francisco, San Francisco, CA 94143

[3]Chan Zuckerberg Biohub – San Francisco, San Francisco, CA 94158

[4]Howard Hughes Medical Institute, University of California San Francisco, San Francisco, CA, 94143

.
† These authors contributed equally to this work.

*Correspondence: yifan.cheng@ucsf.edu

This PDF file includes:

Figures S1 to S10
Tables S1 to S2

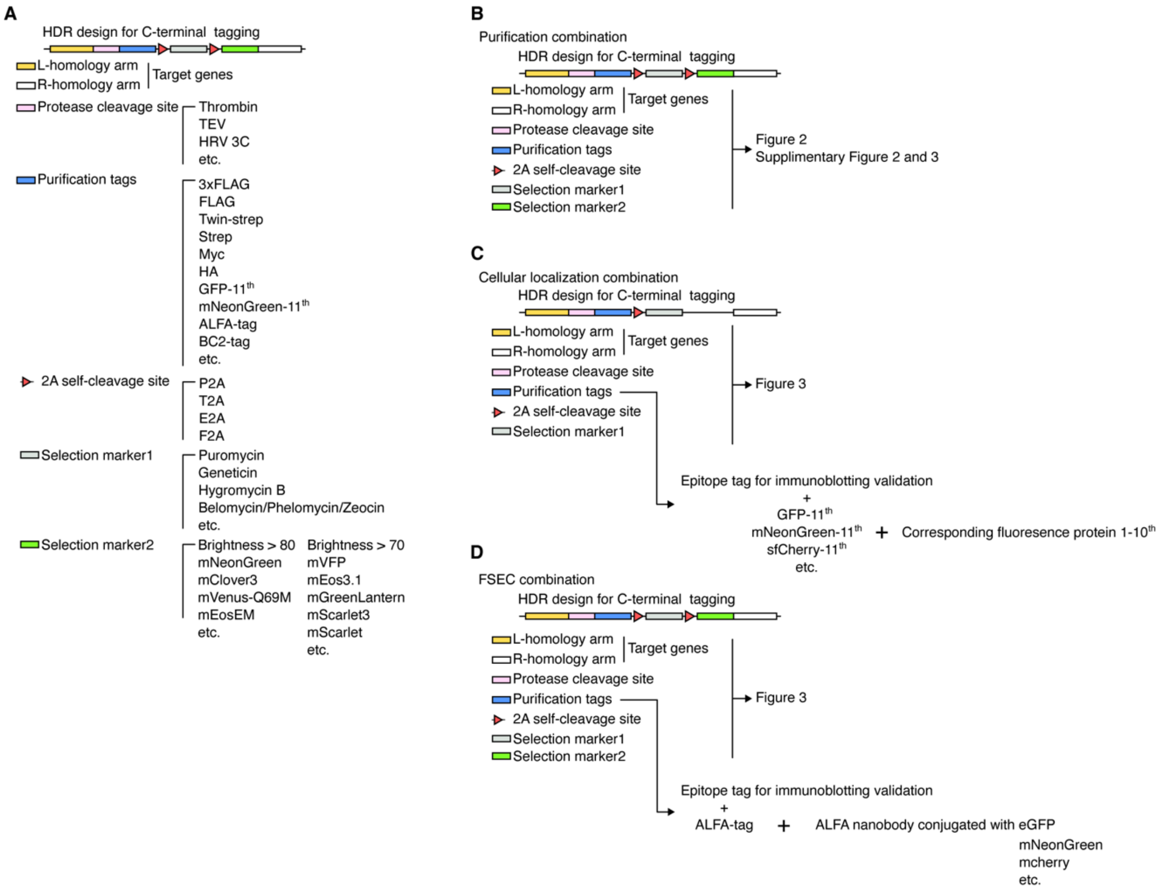

Fig. S1. Summary of variations in different components of pYC for different applications. (A) List of variations in each component of pYC, including protease cleavage site, purification tag, 2A self-cleavage site, antibiotic and fluorescence selection markers. (B-D) Combinations of different components for protein purification (B), cellular localization of target protein by fluorescence microscopy (C), and FSEC (D).

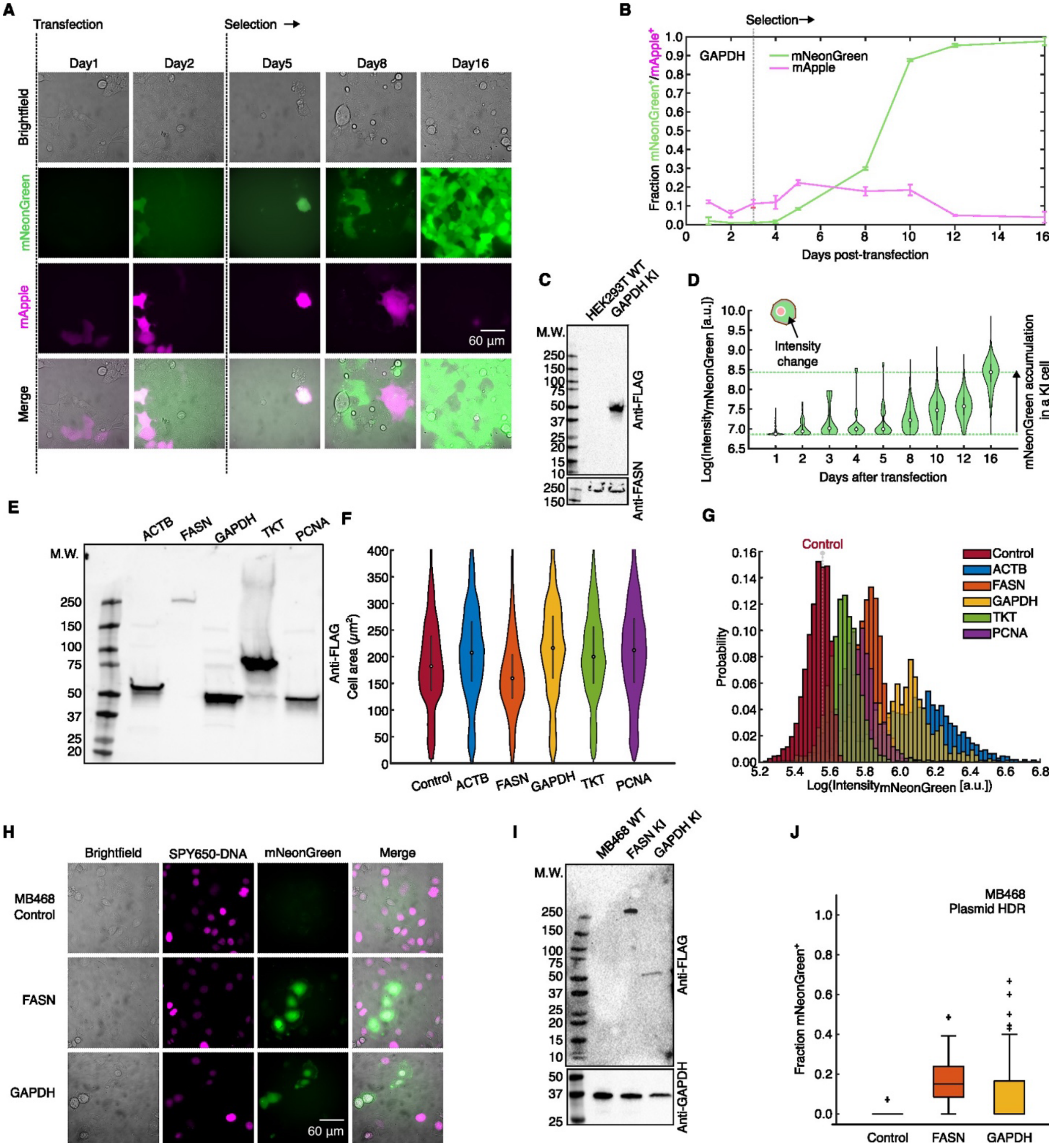

Supplementary Figure 2. Tagging endogenous protein in HEK293T, Jurkat and MDA-MB468 cells. (**A**-**D**) Time courses of fluorescence signals from cultured HEK293T cells after initial transfection to tag endogenous GAPDH. A modified px458-mApple plasmid was used to distinguish fluorescence signals generated from plasmids pYC and px485. **A**: Representative fluorescence microscopy images of bright field (top row), mNeonGreen (second row), mApple (third row) and merged (bottom row), recorded on the indicated days after initial transfection. **B**: Fraction of cells showing Cas9-mApple (magenta) or mNeonGreen (green) fluorescence over

cells. All proteins show their expected size on SDS-PAGE. F: Size distributions of genome-edited cells are comparable to untreated control cells. (Sample number (n) of ACTB, FASN, GAPDH, TKT, PCNA is 2385, 10647, 759, 1912, 2804 cells, respectively) G: Histograms of integrated mNeonGreen fluorescence intensity from genome-edited cells (Sample number (n) of ACTB, FASN, GAPDH, TKT, PCNA is 2385, 10647, 759, 1912, 2804 cells, respectively). Wildtype Jurkat cells (n=3382) were used as control. (H-J) Tagging GAPDH and FASN in MDA-MB468 cells. H: Representative fluorescence images of MDA-MB468 cells. From left to right are bright field image, fluorescence image of cells labeled by SPY650-DNA, fluorescence image of mNeonGreen reveal genome edited cells, and the merged image. I: Tagging in MDA-MB468 is validated by anti-FLAG western blot. J: Knock-in efficiency for FASN and GAPDH in MDA-MB468 cells measured as mNeonGreen signal higher than background signal in untreated control cells.

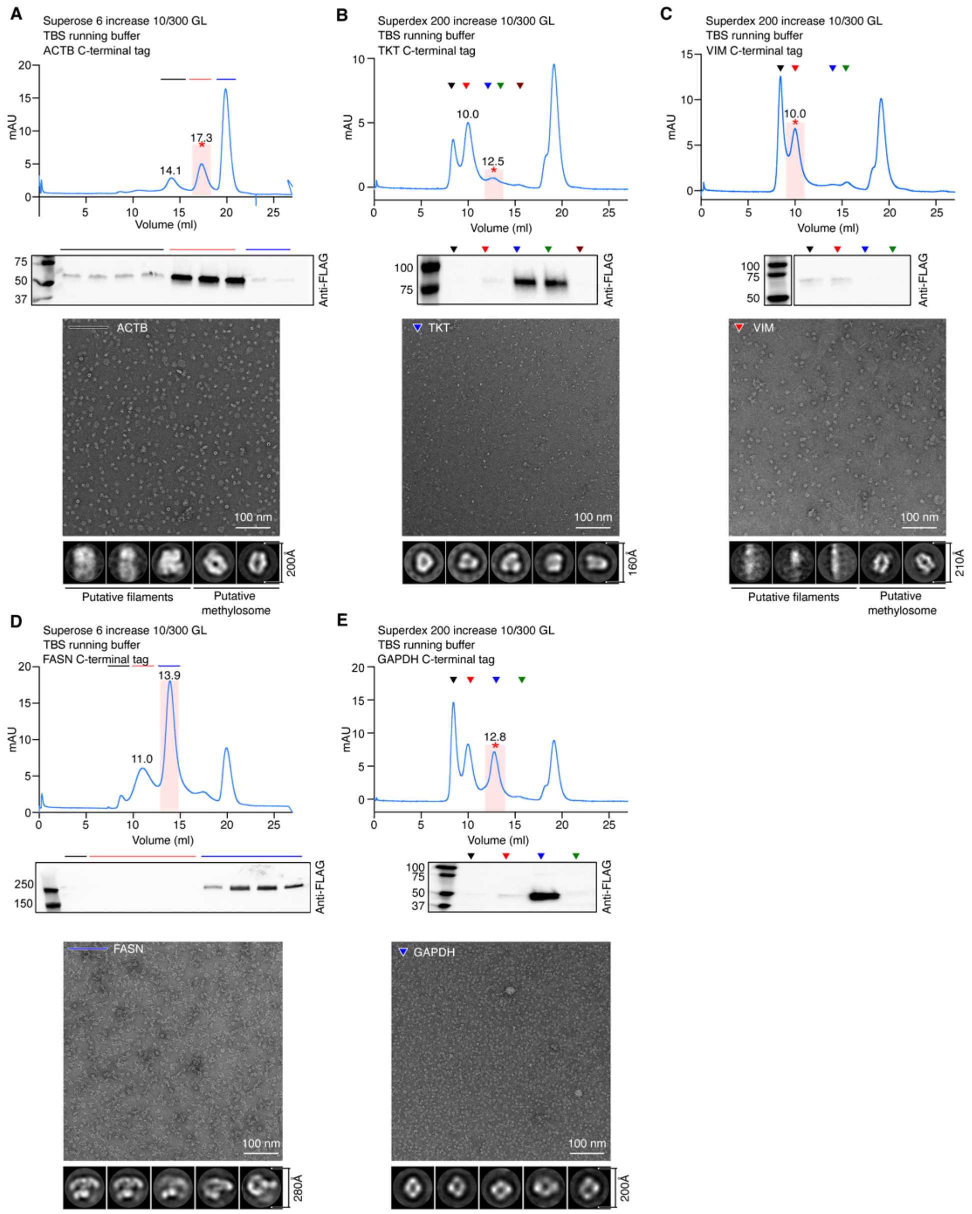

Fig. S3. Purification and negative staining EM of tagged endogenous proteins. Each panel shows a SEC profile of the tagged proteins purified by anti-FLAG M2 resin (top) with colored lines above marking fractions. Anti-FLAG western blots (middle) from each fraction are marked by the same-colored line or triangle. Pink shadow marks the fractions with strongest western blot band. Negative stain EM micrograph and 2D class averages (bottom) of the sample from the fractions marked colored line or triangle on SEC profile. (A) ACTB, (B) TKT, (C) VIM, (D) FASN and (E) GAPDH. Scale bar is 100 nm. Note that SEC profiles often show multiple peaks, indicating that the affinity pulldown captures different complexes associated with the target protein. Furthermore, affinity pulldown could also capture proteins that may not form stable complexes with the target proteins, as we sometimes found other protein in fractions within a SEC peak.

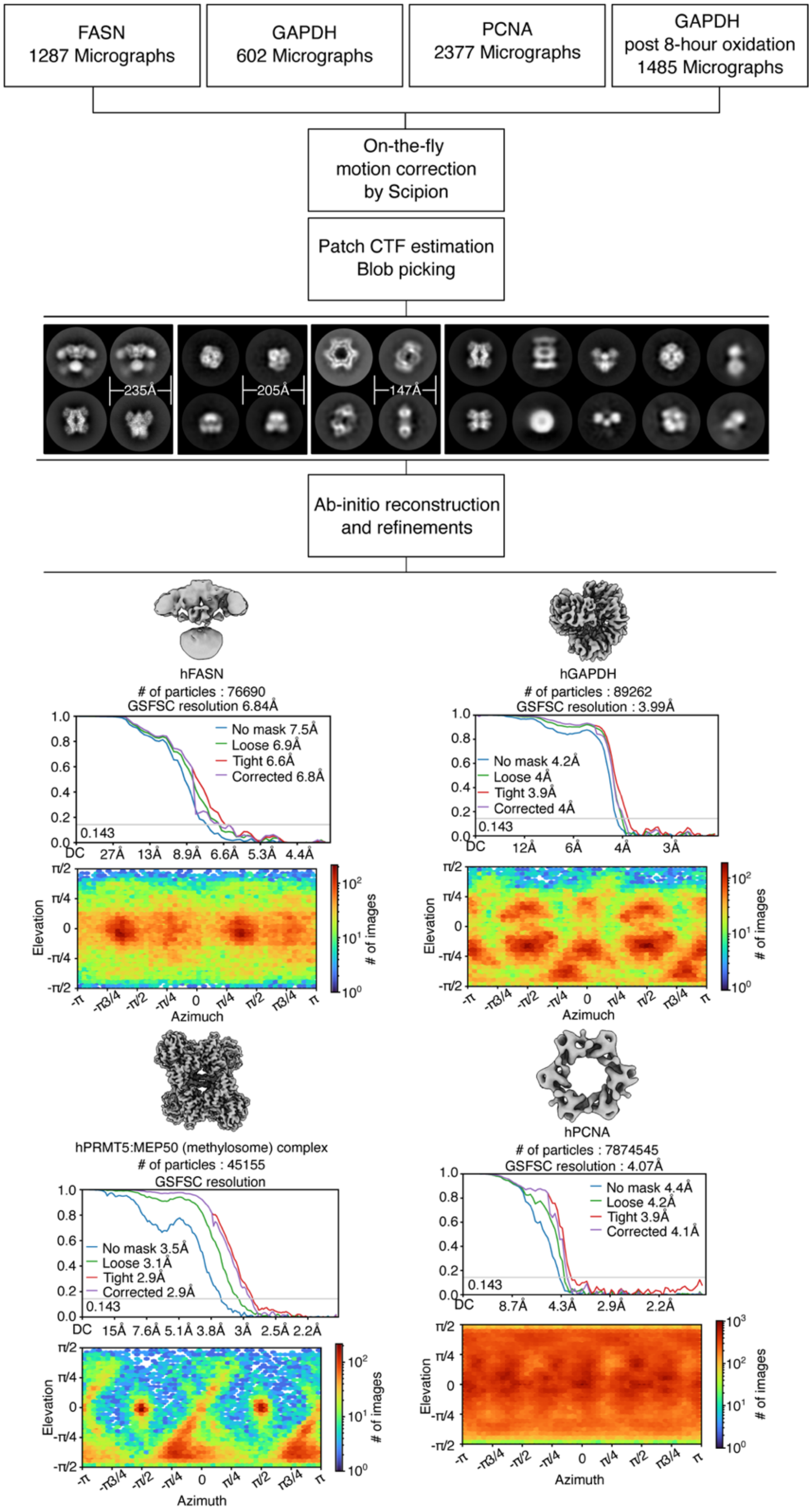

Fig. S4. Image processing workflow. Image processing workflow of 200 kV cryo-EM datasets are illustrated. Reconstructions of FASN, GAPDH, methylosome and PCNA are shown.

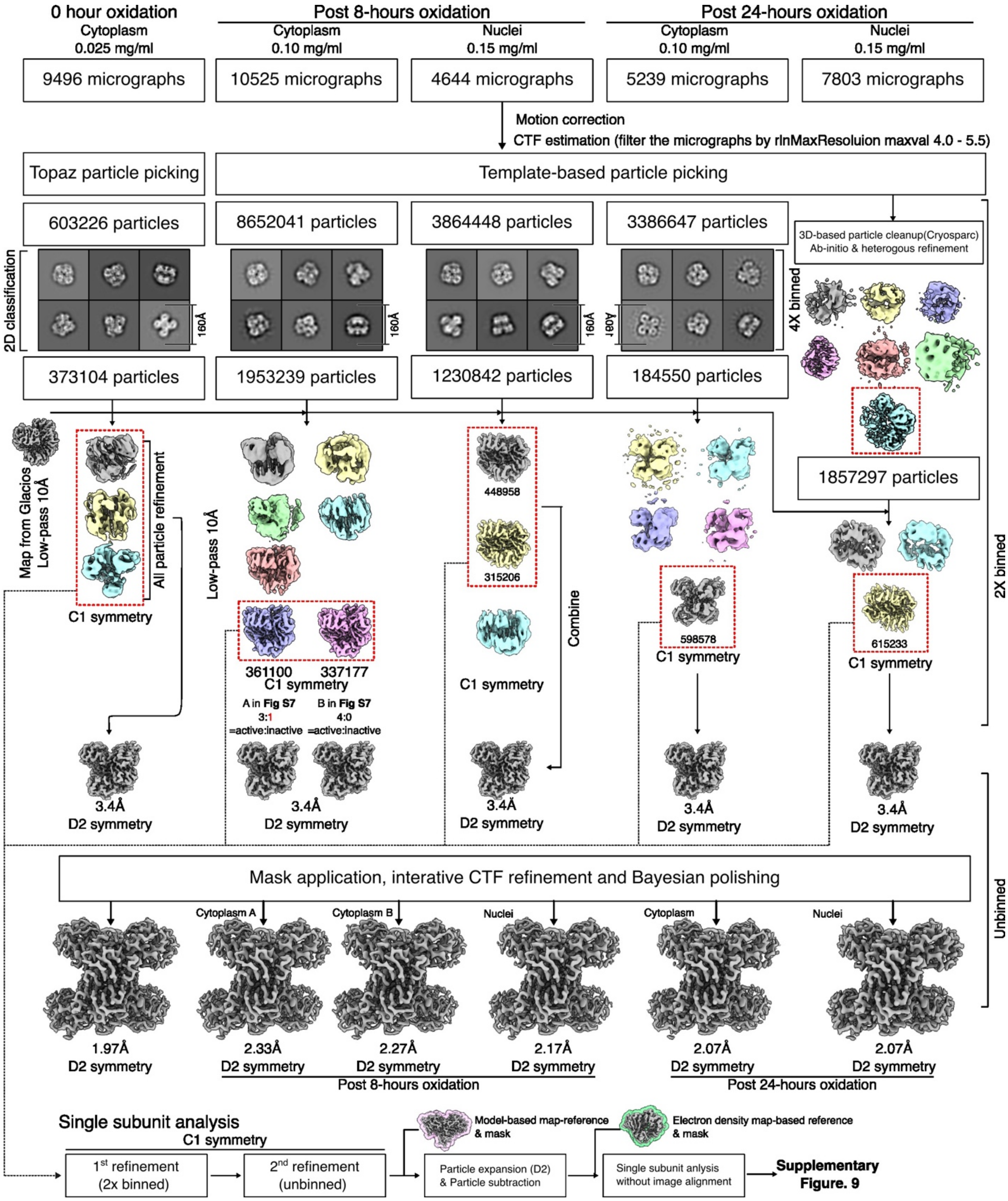

Supplementary Figure 5. The workflow of cryo-EM data processing on analyzing GAPDH. Five cryo-EM datasets of endogenous human GAPDH purified at different time points from cytosol and nucleus of cells after prolonged oxidative stress. The results of single subunits analysis on endogenous GAPDH under the different oxidative environment and cellular compartment further explained in *SI Appendix*, Fig. S9.

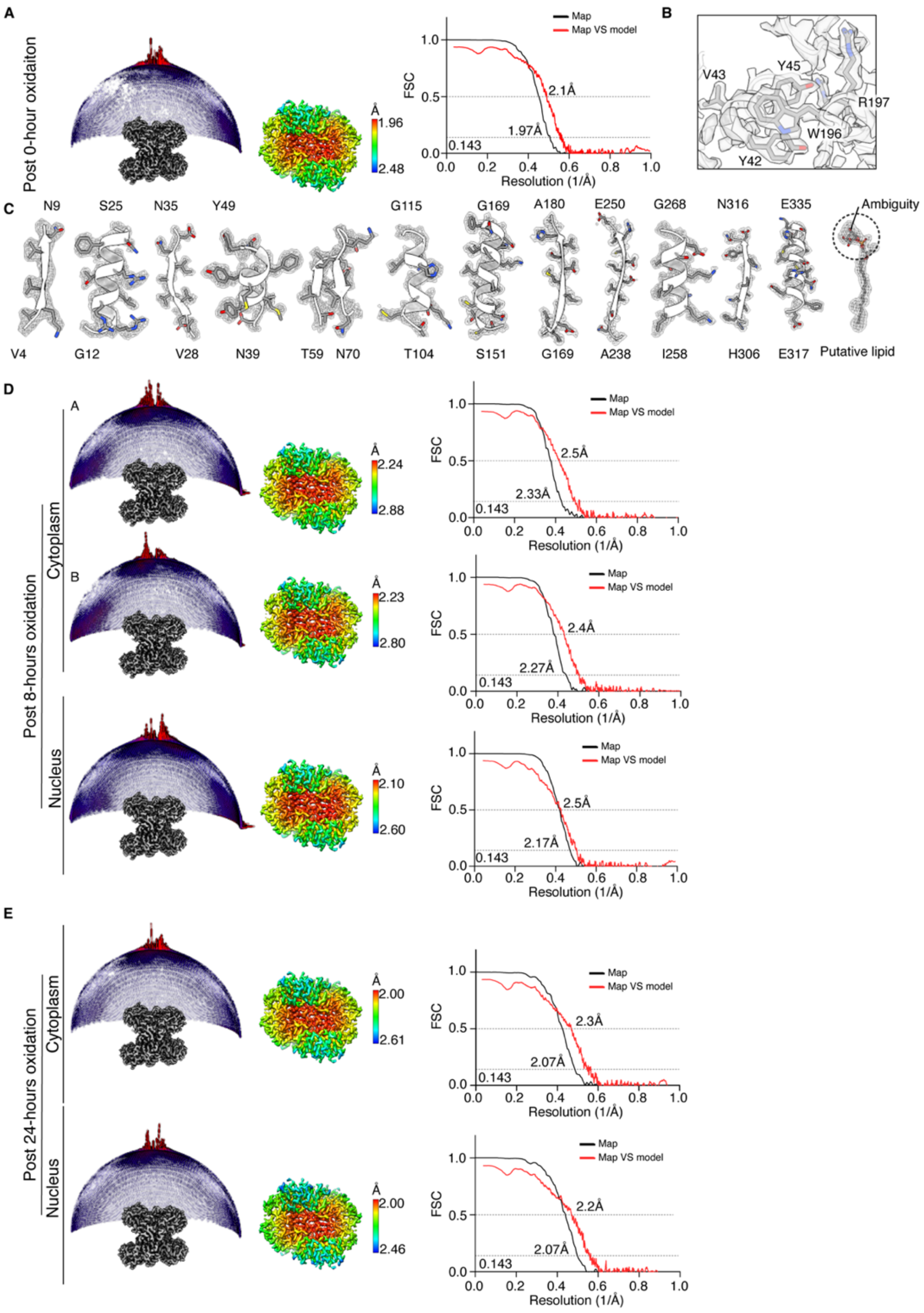

Fig. S6. Cryo-EM structures of endogenous GAPDH. (A) From left to right, angular distribution, local resolution and FSC curves of the endogenous GAPDH from 0-hour oxidation. (B) and (C) Representative densities of human endogenous GAPDH. (D) The three structures, two from cytoplasm and one from nucleus, after 8-hour oxidation are displayed with their angular distribution and location resolution. The FSC curves shows the data quality of cryo-EM density maps and corresponding atomic models. (E) Post 24-hours oxidation GAPDH structures are presented with their angular distribution and local resolution. FSC curves represents the data quality. All statistics data were calculated using Relion.

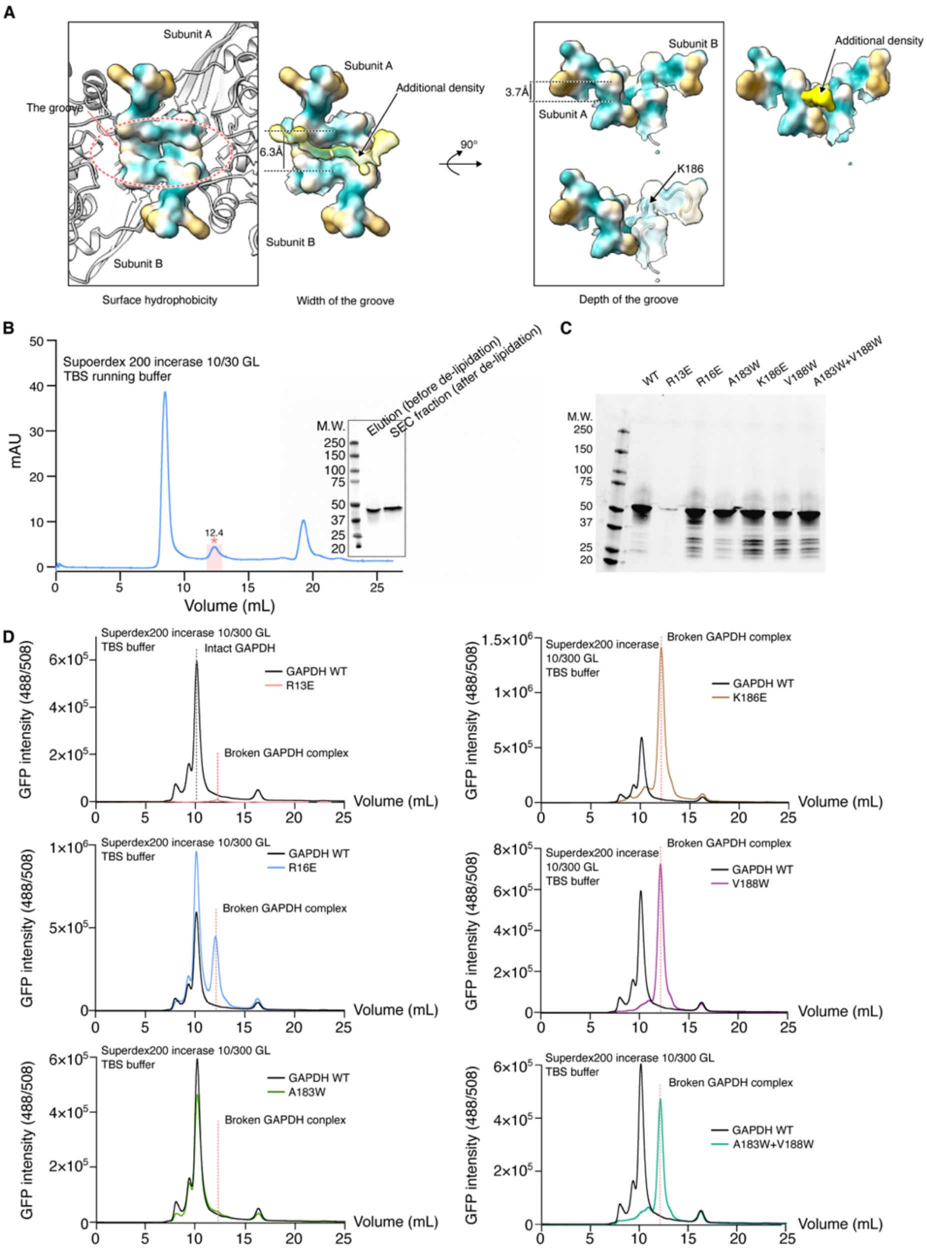

Fig. S7. The lipid-like ligand binding groove in endogenous GAPDH. (A) Hydrophobic surface charge of the groove in endogenous GAPDH. The surface potential is generated by default in ChimeraX. (B) SEC profile of affinity purified GAPDH after de-lipidation treatment. The shaded peak corresponds to the intact tetrameric GAPDH. Insert are anti-FLAG western blot of affinity purified GAPDH before de-lipidation treatment and from the shaded peak after de-lipidation. (C) GFP fluorescence image of SDS-PAGE gel of the lysates from the wild type and mutant GAPDH expressing cells. R13 mutant reduces GAPDH expression. (D) Cell lysate FSEC profiles of wild type and mutant recombinant GAPDH. FSEC profile of cell lysate with wild type GAPDH (black curve) is used as a control showing the location of the intact tetrameric GAPDH (black dashed line). Colored FSEC profiles are from cell lysate of mutant GAPDH. The peak indicated by the red dashed line corresponds to non-intact GAPDH.

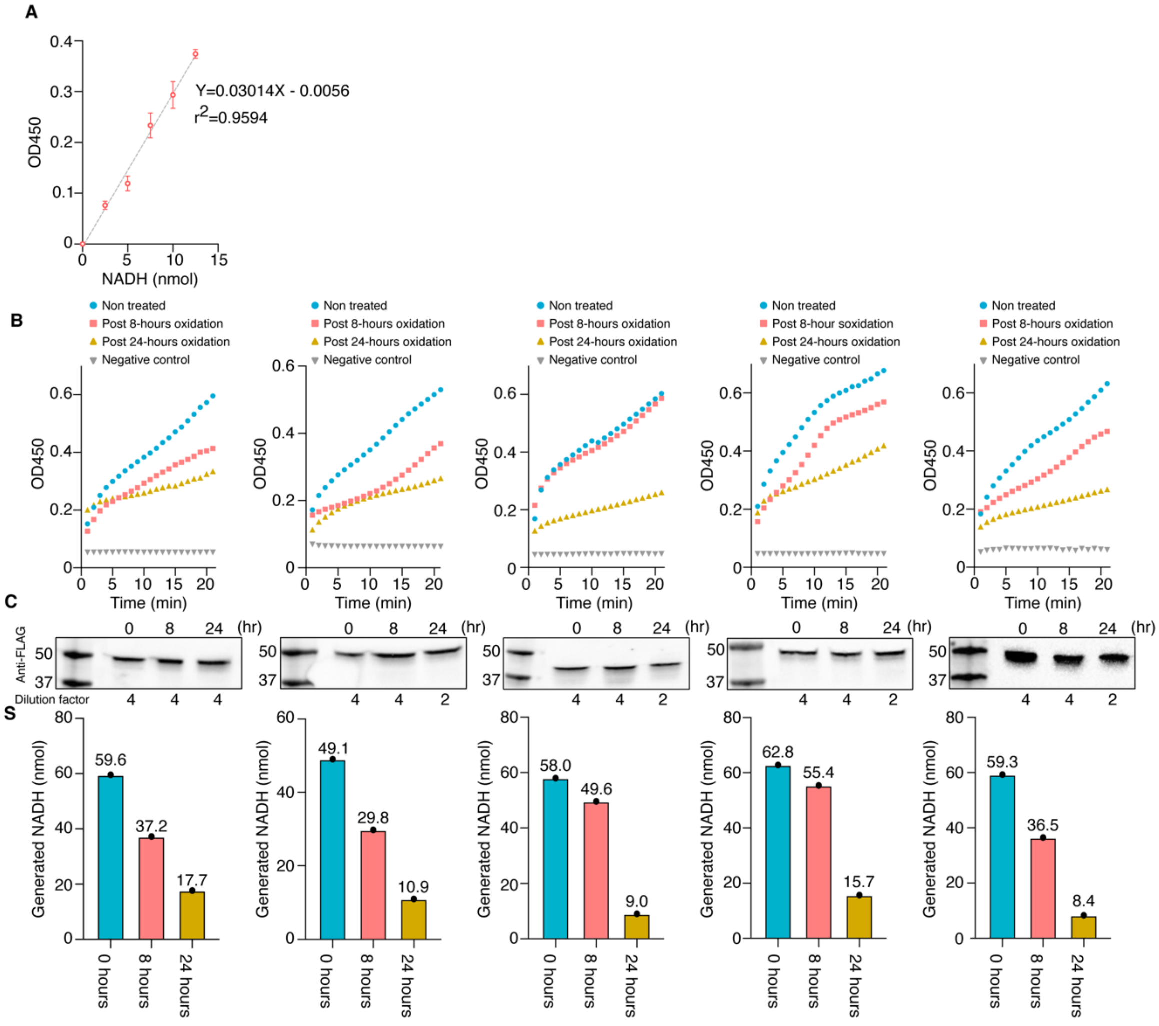

Fig. S8. The enzymatic activity of endogenous GAPDH during oxidation stress. (A) Standard curve of NADH is shown as a reference. (B) Five independent measurements of enzymatic activity are shown during prolonged oxidation stress. (C) Each western blot shows the amount of GAPDH in independent experiments. (D) Generated NADH is calculated from OD450 values. Blue, pink, and yellow indicate 0-, 8-, and 24-hours oxidation stressed sample. Each data point in (B) and (D) shown as a dot.

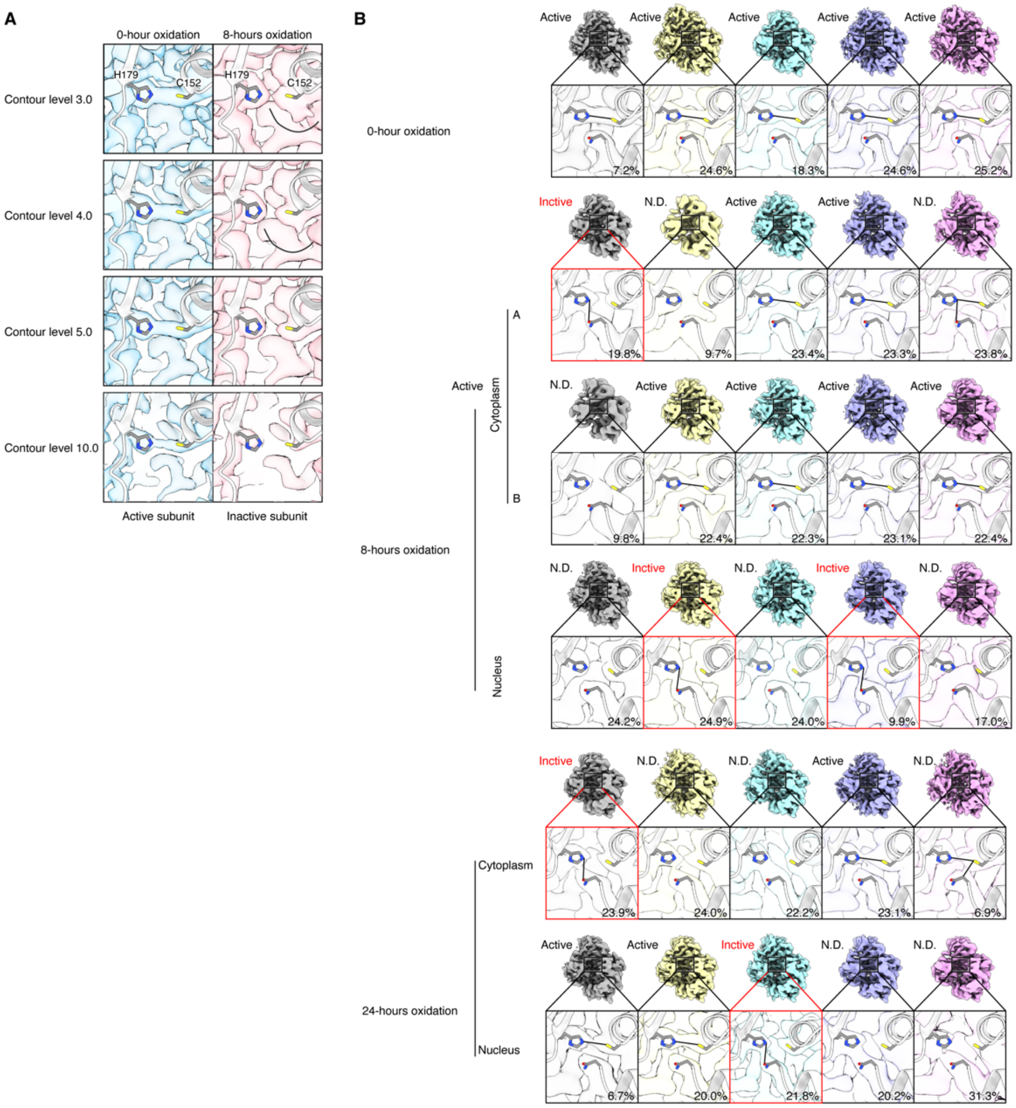

Fig. S9. Catalytic site configurations of endogenous GAPDH. (A) Enlarged view of the catalytic sites of the normalized cryo-EM density maps from cytoplasmic GAPDH without oxidation stress (left) and nuclear GAPDH after 8 hours oxidation (right) displayed at different threshold. (B) Classification of individual subunits without alignment after symmetry expansion. Particles are classified into “Active”, “Inactive” and “N.D.” (not-defined). Percentage of particles within each class are labeled.

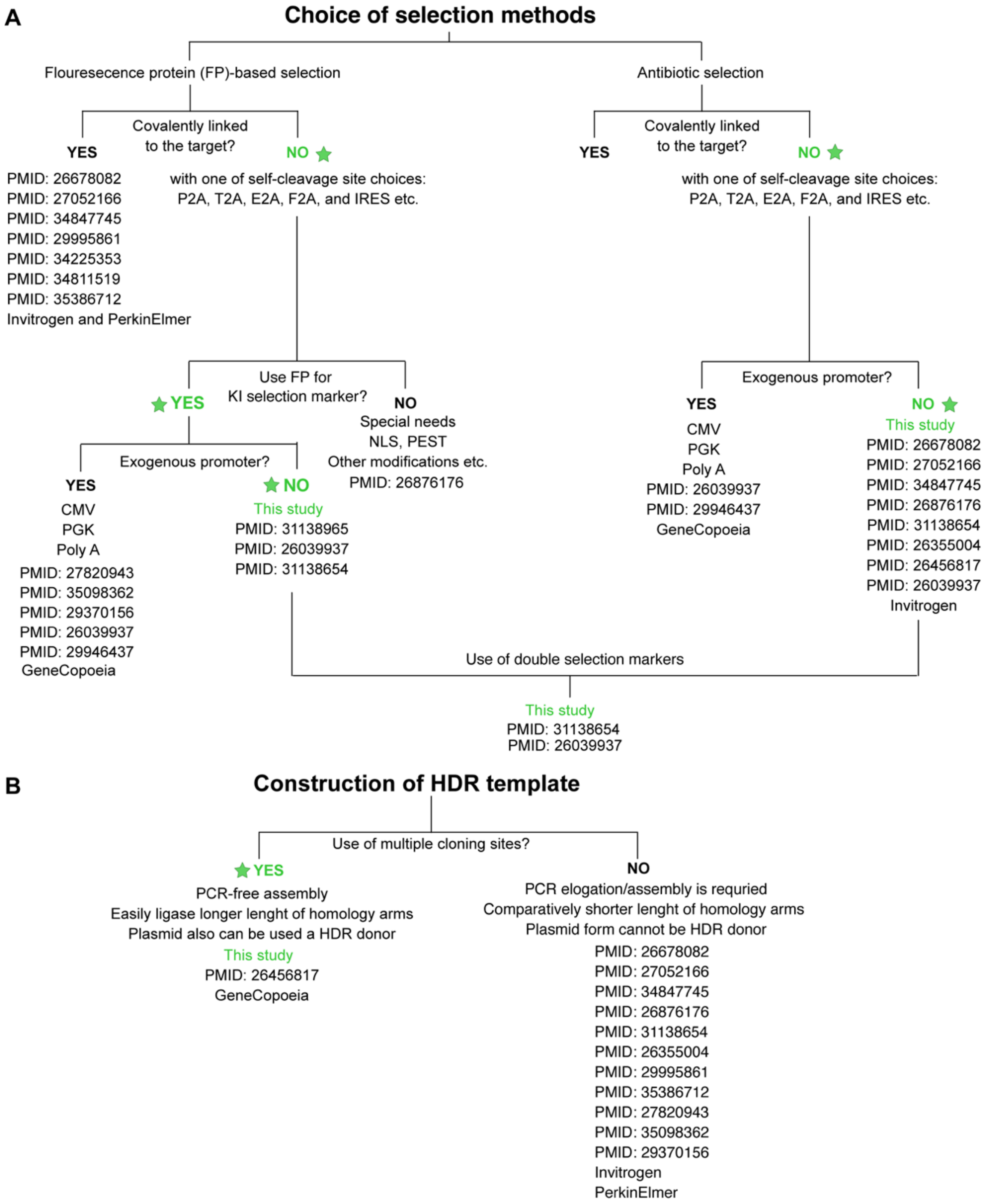

Fig. S10. Comparison of selection strategies and HDR designs. Tree diagrams illustrating different strategies of selection markers and HDR template construction used in different studies or available from vendors. All references are noted with PMID. The strategies used in this study is marked in green. (A) Two commonly selection markers of CRISPR/Cas9 knock-in cells are fluorescent proteins (FP) and antibiotic resistance genes. In our study, we used double selection markers that both are separated from the targeted proteins by the 2A cleavage site. (B) Construction of HDR template. In our study, we used multiple cloning sites (MCS), and the plasmid itself can be used as a HDR donor.

Supplementary Table 1 Cryo-EM data processing and atomic model building of GAPDH

	#1 GAPDH-WT (EMDB-29664) (PDB 8G17)	#2 GAPDH-Cyto8h-Class1 (EMDB-29660) (PDB 8G13)	#3 GAPDH-Cyto8h-Class2 (EMDB-29661) (PDB 8G14)	#4 GAPDH-Nuclear8h (EMDB-29659) (PDB 8G12)	#5 GAPDH-Cyto24h (EMDB-29663) (PDB 8G16)	#6 GAPDH-Nuclear24h (EMDB-29662) (PDB 8G15)
**Data collection and processing**						
Magnification	105,000					
Voltage (kV)	300					
Electron exposure (e–/Å^2)	45.8					
Defocus range (μm)	(-0.8) - (-1.5)					
Pixel size (Å)	0.4175					
Symmetry imposed	D2					
Initial particle images (no.)	603,226	8,652,041	8,652,041	3,386,647	3,864,448	6,440,831
Final particle images (no.)	373,104	361,100	337,177	598,575	764,164	615,233
Map resolution (Å)	1.97	2.33	2.27	2.07	2.17	2.07
FSC threshold	0.143	0.143	0.143	0.143	0.143	0.143
Map resolution range (Å)	1.96-2.48	2.24-2.88	2.23-2.80	2.10-2.60	2.00-2.61	2.00-2.46
**Refinement**						
Initial model used (PDB code)	4WNC					
Model resolution (Å)	2.0	2.4	2.3	2.1	2.3	2.1
FSC threshold	0.5	0.5	0.5	0.5	0.5	0.5
Map sharpening *B* factor (Å^2)	-49.63	-69.10	-70.25	-53.81	-68.56	-30.20
Model composition						
Non-hydrogen atoms	10,016	10,008	10,020	10,108	9,980	10,056
Protein residues	1,336	1,336	1,336	1,336	1,336	1,336
Ligands	0	0	0	0	0	0
B factors (Å^2)						
Protein	100.29	132.60	127.28	103.30	148.43	94.07
Ligand	N/A	N/A	N/A	N/A	N/A	N/A
R.m.s. deviations						
Bond lengths (Å)	0.005	0.004	0.005	0.005	0.006	0.005
Bond angles (°)	0.976	0.597	0.720	1.092	1.082	1.09
Validation						
MolProbity score	1.53	1.59	1.33	1.59	1.61	1.81
Clashscore	5.82	5.94	2.41	4.86	5.72	5.30
Poor rotamers (%)	0.76	0.86	1.13	1.20	0.38	1.69
Ramachandran plot						
Favored (%)	96.69	96.08	96.08	95.93	95.74	94.94
Allowed (%)	3.31	3.92	3.92	4.07	4.26	5.06
Disallowed (%)	0.00	0.00	0.00	0.00	0.00	0.00

Table S2. Sequence of sgRNA and homology arms of six genes targeted in this study.

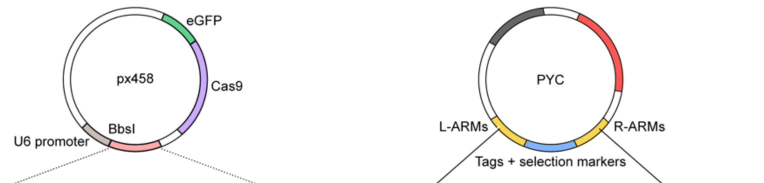

ACTB:AGTCCGCCTAGAAGCATTTG
FASN : CCGGTGGCAGGCGGGGGCAC
GAPDH : CCTCCAAGGAGTAAGACCCC
TKT : CCACACTTCATACCCGCCCT
VIM : GTGTGTGCAATTTTTATTCA
PCNA : CGAGGATGAAGAAGGATCTT

ACTB

GCGGCCGCGTATACATGAGGGTTACCCCTCG
GGGCTGTGCTGTGGAAGCTAAGTCCTGCCCT
CATTTCCCTCTCAGGCATGGAGTCCTGTGGCA
TCCACGAAACTACCTTCAACTCCATCATGAAG
TGTGACGTGGACATCCGCAAAGACCTGTACG
CCAACACAGTGCTGTCTGGCGGCACCACCAT
GTACCCTGGCATTGCCGACAGGATGCAGAAG
GAGATCACTGCCCTGGCACCCAGCACAATGA
AGATCAAGGTGGGTGTCTTTCCTGCCTGAGCT
GACCTGGGCAGGTCGGCTGTGGGGTCCTGT
GGTGTGTGGGGAGCTGTCACATCCAGGGTCC
TCACTGCCTGTCCCCTTCCCTCCTCAGATCAT
TGCTCCTCCTGAGCGCAAGTACTCCGTGTGG
ATCGGCGGCTCCATCCTGGCCTCGCTGTCCA
CCTTCCAGCAGATGTGGATCAGCAAGCAGGA
GTATGACGAGTCCGGCCCCTCCATCGTTCATC
GCAAATGCTTCTTCGAATCTAGA PAM site mutation

GAATTCTAGGCGGACTATGACTTAGTTGCGTTA
CACCCTTTCTTGACAAAACCTAACTTGCGCAGA
AAACAAGATGAGATTGGCATGGCTTTATTTGTT
TTTTTTGTTTTGTTTTGGTTTTTTTTTTTTTTTG
GCTTGACTCAGGATTTAAAAACTGGAACGGTG
AAGGTGACAGCAGTCGGTTGGAGCGAGCATC
CCCCAAAGTTCACAATGTGGCCGAGGACTTTG
ATTGCACATTGTTGTTTTTTTAATAGTCATTCCA
AATATGAGATGCGTTGTTACAGGAAGTCCCTTG
CCATCCTAAAAGCCACCCCACTTCTCTCTAAGG
AGAATGGCCCAGTCCTCTCCCAAGTCCACACA
GGGGAGGTGATAGCATTGCTTTCGTGTAAATTA
TGTAATGCAAAATTTTTTTAATCTTCGCCTTAATA
CTTTTTTATTTTGTTTTATTTTGAATGATGAGCCT
TCGTGCCCCCCCTTCCCCCTTTTTTGTCCCCC
AACTTGAGATGTATGAAGGCTTTTGGTCTCCCT
GGGAGTGGGTGGAGGCAGCCAGGGCTTACCT
GTACACTGACTTGAGACCAGTTGAATAAAAGTG
CACACCTTAAAAATGAGGCCAAGTGTGACTTTG
TGGTGTGGCTGGGTTGGGGGCAGCAGAGGGT
GAACCCTGCAGGAGGGTGAACCCTGCAAAAG
GGTGGGGCAGTGGGGGCCAACTTGTCCTTAC
CCAGAGTGCAGGTGTGTGGAGATCCCTCCTGC
CTTGACATTGAGCAGCCTTAGAGGGTGGGGGA
GGCTCAGGGGTCAGGTCTCTGTTCCTGCTTAT
TGGGGAGTTCCTGGCCTGGCCCTTCTATGTCT
CCCCAGGTACCCCAGTTTTTCTGGGTTCACCC
AGAGTGCAGATGCTTGAGGAGGTGGGAAGGG
ACTATTTGGGGGTGTCTGGCTCAGGTGCCATG
CCTCACTGGGGCTGGTTGGCACCTGCATGTAT
ACAAGCTTCTCGAG

FASN

GCGGCCGCGTATACCCGCAGGACCCAGAGTA
CTGGGCAGGAACGGAAGGGGGAAGGGAAGG
ATGGGATGGGACGCGAAGAGCGTTAAAGGCC
CCCCAACTTCACCCCTTTAATAGCCGATCCCG
GCCGGATGCGGTTGCTCATGCCTGTAATCCC
GGCACTTTGGGAGGCTAAGGCGGGCAGATTA
CCTGAGGTCGGGAGTTCGAGACCAGCCTGAC
CAACGTGGAGAAACCCCTTCTCTACTAAAAAT
ACAAAAATTAGCCAGGCATGGTGGCACTCACC
TATAGTCCCACCTACTCGGGAGGAGAATCGCT
TGAACCGGGTAGGTGGAGGTTGCGGAGGGA
GGAGAATCGCTTGAACCGGGTAGGTGGAGGT
TGCGGTGAGCTGAGATCGCCCCACTCCATCT
AGCCTGGGTAACAAAAATGAAACTCGTCTCAA
AAAAAAAAAGCCCACCCGCAGTGTAGCACAG
GGCAGGCCGGTGCTGTGGGTGCTGCCAGCA
CCTGCTCAGAGCCCGACCCCACAGGTGCTGG
AGGCGCTGCTGCCGCTGAAGGGCCTAGAGG
AGCGTGTGGCAGCCGCCGTGGACCTGATCAT
CAAGAGCCACCAGGGCCTGGACCGCCAGGA
GCTGAGCTTTGCGGCCCGGTCCTTCTACTAC
AAGCTGCGTGCCGCTGAGCAGTACACACCCA
AGGCCAAGTACCATGGCAACGTGATGCTACTG
CGCGCCAAGACGGGTGGCGCCTACGGCGAG
GACCTGGGCGCGGACTACAACCTCTCCCAGG
TGCGCAAGGGGGCCTGACGGGAACGGGGACA
GGGACAGGAGTGGGGTGGGACGGCAGCTGA
TCGCATCCCCTGCAGGTATGCGACGGGAAAG
TATCCGTCCACGTCATCGAGGGTGACCACCG
CACGCTGCTGGAGGGCAGCGGCCTGGAGTC
CATCATCAGCATCATCCACAGCTCCCTGGCTG
AGCCACGCGTGAGCGTGCGGGAGTTCGAATC
TAGA

GAATTCTAGGCCTGTGCCCCCGCCTGCCACC
GGAGGTCACTCCACCATCCCCACCCCACCCC
ACCCCACCCCCGCCATGCAACGGGATTGAAG
GGTCCTGCCGGTGGGACCCTGTCCGGCCCAG
TGCCACTGCCCCCCGAGGCTGCTAGATGTAG
GTGTTAGGCATGTCCCACCCACCCGCCGCCTC
CCACGGCACCTCGGGGACACCAGAGCTGCCG
ACTTGGAGACTCCTGGTCTGTGAAGAGCCGG
TGGTGCCCGTGCCCGCAGGAACTGGGCTGGG
CCTCGTGCGCCCGTGGGGTCTGCGCTTGGTC
TTTCTGTGCTTGGATTTGCATATTTATTGCATTG
CTGGTAGAGACCCCCAGGCCTGTCCACCCTG
CCAAGACTCCTCAGGCAGCGTGTGGGTCCCG
CACTCTGCCCCCATTTCCCCGATGTCCCCTGC
GGGCGCGGGCAGCCACCCAAGCCTGCTGGC
TGCGGCCCCCTCTCGGCCAGGCATTGGCTCA
GCCCGCTGAGTGGGGGGTCGTGGGCCAGTC
CCCGAGGAGCTGGGCCCCTGCACAGGCACAC
AGGGCCCGGCCACACCCAGCGGCCCCCCGC
ACAGCCACCCGTGGGGTGCTGCCCTTATGCC
CGGCGCCGGGCACCAACTCCATGTTTGGTGT
TTGTCTGTGTTTGTTTTTCAAGAAATGATTCAAA
TTGCTGCTTGGATTTTGAAATTTACTGTAACTG
TCAGTGTACACGTCTGGACCCCGTTTCATTTTT
ACACCAATTTGGTAAAAATGCTGCTCTCAGCCT
CCCACAATTAAACCGCATGTGATCTCCAGTATA
CAAGCTTCTCGAG

GAPDH

GTATACAGCAATGCCTCCTGCACCACCAACTGCTTAGCA
CCCCTGGCCAAGGTCATCCATGACAACTTTGGTATCGTG
GAAGGACTCATGGTATGAGAGCTGGGGAATGGGACTGA
GGCTCCCACCTTTCTCATCCAAGACTGGCTCCTCCCTGC
CGGGGCTGCGTGCAACCCTGGGGTTGGGGGTTCTGGG
GACTGGCTTTCCCATAATTTCCTTTCAAGGTGGGGAGGG
AGGTAGAGGGGTGATGTGGGGAGTACGCTGCAGGGCC
TCACTCCTTTTGCAGACCACAGTCCATGCCATCACTGCC
ACCCAGAAGACTGTGGATGGCCCCTCCGGGAAACTGTG
GCGTGATGGCCGCGGGGCTCTCCAGAACATCATCCCTG
CCTCTACTGGCGCTGCCAAGGCTGTGGGCAAGGTCATC
CCTGAGCTGAACGGGAAGCTCACTGGCATGGCCTTCCG
TGTCCCCACTGCCAACGTGTCAGTGGTGGACCTGACCT
GCCGTCTAGAAAAACCTGCCAAATATGATGACATCAAGA
AGGTGGTGAAGCAGGCGTCGGAGGGCCCCCTCAAGGG
CATCCTGGGCTACACTGAGCACCAGGTGGTCTCCTCTG
ACTTCAACAGCGACACCCACTCCTCCACCTTTGACGCT
GGGGCTGGCATTGCCCTCAACGACCACTTTGTCAAGCT
CATTTCCTGGTATGTGGCTGGGGCCAGAGACTGGCTCTT
AAAAAGTGCAGGGTCTGGCGCCCTCTGGTGGCTGGCTC
AGAAAAAGGGCCCTGACAACTCTTTTCATCTTCTAGGTAT
GACAACGAATTTGGCTACAGCAACAGGGTGGTGGACCT
CATGGCCCACATGGCCTCCAAAGAGTTCGAATCTAGA

PAM site
slience mutation

GAATTCTAAGACCCCTGTACCACTAGCCCCAGCAAGAG
CACAAGAGGAAGAGAGAGACCCTCACTGCTGGGGAGT
CCCTGCCACACTCAGTCCCCCACCACACTGAATCTCCC
CTCCTCACAGTTGCCATGTAGACCCCTTGAAGAGGGGA
GGGGCCTAGGGAGCCGCACCTTGTCATGTACCATCAAT
AAAGTACCCTGTGCTCAACCAGTTACTTGTCCTGTCTTA
TTCTAGGGTCTGGGGCAGAGGGGAGGGAAGCTGGGCT
TGTGTCAAGGTGAGACATTCTTGCTGGGGAGGGACCT
GGTATGTTCTCCTCAGACTGAGGGTAGGGCCTCCAAAC
AGCCTTGCTTGCTTCGAGAACCATTTGCTTCCCGCTCA
GACGTCTTGAGTGCTACAGGAAGCTGGCACCACTACTT
CAGAGAACAAGGCCTTTTCCTCTCCTCGCTCCAGTCCT
AGGCTATCTGCTGTTGGCCAAACATGGAAGAAGCTATT
CTGTGGGCAGCCCCAGGGAGGCTGACAGGTGGAGGA
AGTCAGGGCTCGCACTGGGCTCTGACGCTGACTGGTT
AGTGGAGCTCAGCCTGGAGCTGAGCTGCAGCGGGCAA
TTCCAGCTTGGCCTCCGCAGCTGTGAGGTCTTGAGCA
CGTGCTCTATTGCTTTCTGTGCCCTCGTGTCTTATCTGA
GGACATCGTGGCCAGCCCCTAAGGTCTTCAAGCAGGAT
TCATCTAGGTAAACCAAGTACCTAAAACCATGCCCAAGG
CGGTAAGGACTATATAATGTTTAAAAATCGGTAAAAATGC
CCACCTCGCATAGTTTTGAGGAAGATGAACTGAGATGT
GTCAGGGTGACTTATTTCCATCATCGTCCTTAGGGGAAC
TTGGGTAGGGGCAAGGCGTGTAGCTGGGACCTAGGTC
CAGACCCCTGGCTCTGCCACTGAACGGCTCAGTTGCT
TTGGGCAGTTACTCCCGGGCCTCACTTTGCACGTGTGC
TGTATACAAGCTTCTCGAG

TKT

GCGGCCGCGTATACGGGCAAGGGACAGGGCCAGAGA
GCCGAGCGTTGGACGTGCCTCTTCTATCTGAGCCCCA
GGAGGGCAGCTGTGGCCCAGAACACCCAGAGCTGTC
CCTCAGAGCAGCTGGGACGTGGTCTCTTAAAAGGCCT
CAGGCTTGGCCTGGGGACCTAGCTAACTCACAGCTGC
TGATGAGCTCTGTGGAGCTCCTGCTGCCCAGGAAGCT
GGTGAGGATTCCAACCTCGCTGCTAAATGGTGGATGGT
GGCGTCCCTGAGGTAGTGTAGGGTGGAAGCTATGGTG
CAGCTTGGGAAGCAATGCAGAATTTCTGGTATTTGTGA
GTCCTTCACACAGCAGGCTCCCTAAGTACACACCAGCC
TCTCTAAGTAGGAGGCCCCAGTGGGAGAGATGGGCTT
TGACTCTGGGGTCAAATGTAGATAATTGGACTATGGACA
GTGGCTGGCTGGTCACCAACAATGGTGTTTGAAACAAA
CATTTAGAGGCCATATTTGGGCTTATAAAAATAGTTCTGG
GCCGTGCATGGTGGCTCACACCTGCAATCCCAGCACT
TTGGGAGGCTGAGGACAGCGGATTTCTTGAGCTCAGG
AGTTGGGAGACCAGCCTGGGCAACATGGTGATACCTG
TCTGTCTCTTTAAAATAAAAAAAATCAATGAGTTATGTGA
TGGGCTCATGGCTACAGGTGGAGAAAGGCAGTGCATAT
GCAGCCTCCTCCATCCTTGACTAAGGCTGACAGAGGG
CTGGGCCCACCACTGCTCACCCTGAGGCCTCGTCTTC
TGACTCCCCTCCTTTCATTTCTAGGTGGCATTGGTGAG
GCTGTGTCCAGTGCAGTAGTGGGCGAGCCTGGCATCA
CTGTCACCCACCTGGCAGTTAACCGGGTACCAAGAAGT
GGGAAGCCGGCTGAGCTGCTGAAGATGTTTGGTATCG
ACAGGGATGCCATTGCACAAGCTGTGAGGGGCCTCAT
CACCAAGGCCTTCGAATCTAGA

GAATTCTAGGGCGGGTATGAAGTGTGGGGCGGGGGTC
TATACATTCCTGAGATTCTGGGAAAGGTGCTCAAAGATG
TACTGAGAGGAGGGGTAAATATATGTTTTGAGAAAAATG
AATTGGCCCTGAAATGTTTTCTTTTTTGATGTGTTTGCA
GCCAGTGCAGGTTCTGGGGAAGAAGCGTGGAGGGTG
CGCTAGGGAGGCTGGTGTCCTTGGTCCTGGGCCAGGA
CCTGGGTGTCCCAAGTCCCCTTGGAATCACCTGCAACT
GCCCTCAACCTCCAGAACTATCGCTGCCTCCCATTCAT
CACCAGGTGACATTTGACTGCAACCTGCCTTCTAGCTG
AGAGGCTGAGACCTACATCCCTCATTGTGACCTCAGTC
CACCTGGCCCTGAGCGGGCTGGGGAACTGCCTCAGTC
TGGAAGCTGACCAGGCACTCTCAGGGCCGCCCCACCT
CCCCCAAGTCCCCACAGCCTTGCAGTCAGGTCTCCTG
GGATAGGGAGGTTCACTTGCTTGTTGTCCCTCGTCCTT
GTCATATCCTTTTAACTAGGCATCTCAGAGAAGCAGAGA
CAGGGCAGCCTTCGTCCTGGGGGAAAAGGGACCCTCA
GGATGGCATGAGAGGTCCTCAATCCCAAGTGTGGAACT
GTCCCCCTCAACTTGTTAAAATGCAGATTTCTGGGTCTT
GCCAATGGGGCCTGGGACTCCATGTGACAACTGGCCC
AGGAGCTTCTGATGTCACACAGATTTCTGCAGTCCCAA
GCTCCAGCCCCGACCTGCTCTGCTGTTCCTAGGTGAC
TGCCCTCACACTGCTGACCACAGTGGATTTCTCCCCCT
GCTGCTCGGGCTCAGCTGGGGTCAGCCCTGCTTATAA
GGTCAACTGTGCAAAACCTTATACTGGCCAAGAACAAA
CTAGTGCTGGGGGAGGAGGGCTGGGTGCCCCGGCCA
CTGGTGGAGTCCCCAGGAAATCCTCAGAGCTGTTGCG
AGGATGAGACAGTATACAAGCTTCTCGAG

VIM

GCGGCCGCGTATACTAGGGTAATCTCAGACAGGAGTT
GATATATTTTAAAATCAGTGAATCTGAATCTCAGATACAG
CTGGCTAATTTGAGAGGTTCAGGTTTCATTCATGCCTA
CTAAAAAAAGAATAGGCTTCTTCTTCCAGCAGTACACA
CAGCCAACTAATTATTTGGCTCCTGGATGTGAAGTTGA
GATAGCAGTCTTCCTGTGCTCCAGAATTAGTGATTTGC
TTTGGTGCTTAATTTGAAGTGGGAGTAAGCTTCCTTAAA
CCACTTCCTAAAGCAGCTACATGAAACAGCTTCACTAG
ACTACCTCAATATGAGGAATGTTTTGATCCTGGACATAT
GGTGTCTTCCTACCTCCATACTTTATAGATTCCTAAACC
CATCTATATAATACAAGCATGTGCCATACGATCATTTAGT
TTCTTATTACCTCCCTATGCCAGGAAAGAAATAGTTGCA
ATTTATTGTAGTCATCATGAAATCTTCCCTTGCACATAAA
TTTAAAATGTACCTGCTGCACATTTTAATATGTCTTAATT
GCTTTTAAACTTGGCTGTATTGTGTACAACTATTATACCA
TCTTTTATAAACACAGTTTTTTAAGAAATTTCTTTTTGTA
AGTTACAACATTCCACTGGATCCTTATATTGCCTGTAGT
GGAAGAGGGTCTTGTGTGTCTGCCCCTTCTAGTTTTCA
CTCATGCAGAAGCAACATAACCTTCTGATTTGCACAATA
AATTACATATATTTAGCAGGATTTTTATTTGCCGTGATATA
TAGGATAATTTAGTCTTTGGCATGTGGCATTATATTTATT
TTGGTTTTTTTTTTTTAAACAGGTTATCAACGAAACTTCT
CAGCATCACGATGACCTTGAATTCGAATCTAGA

GAATTCTAAAAATTGCACACACTCAGTGCAGCAATATAT
TACCAGCAAGAATAAAAAAGAAATCCATATCTTAAAGAA
ACAGCTTTCAAGTGCCTTTCTGCAGTTTTTCAGGAGCG
CAAGATAGATTTGGAATAGGAATAAGCTCTAGTTCTTAA
CAACCGACACTCCTACAAGATTTAGAAAAAAGTTTACAA
CATAATCTAGTTTACAGAAAAATCTTGTGCTAGAATACTT
TTTAAAAGGTATTTTGAATACCATTAAAACTGCTTTTTTTT
TTCCAGCAAGTATCCAACCAACTTGGTTCTGCTTCAATA
AATCTTTGGAAAAACTCTTTTGTTGTGTTATTTATTGGAT
AATATCTAAACAATTCTCTACTTGGTCCTATTAGTTAATTT
GTCATTACAATCATGTAAGTTGATAAATTCAGGTTATTTA
TGCTTGAGATGTAGTTCTTAATTTTGTCATTTTTGATAGA
CCTCACTTCTTTTTATTATTACTTAAAAACATTTACAAATA
GGTGGTGTCGAATAAAATAACTTGTACCAAAATGAAGAT
AGGTCTCTCTAAAATGAGCTCAGGTCTGTGATTTTAATC
ATAACAAACAGACTTCCTAAAATTAAAAAATAAAAACTTT
TTTTAGTACATATAGCATATGAGTAACACAAATTCCACTT
TGGGAGTTAAAGCATAGGAAGTTGCCAAGATATAGGGG
CTTATCTTCCGCTAGCAAGATGCAGAGAAATGGAAAAA
GTTCACCAAGTTTTTCTTTTATTTAAGACAGGATCTCAC
TTTGTCACTCAAGCTGGAGTAAAGTGACTCAATCATAGT
TCACTGCAGCCAACAACTCCTAGGCTCAGGCAATCCTC
CCACCTCAGCCTCCTCAGTAGCTAGGAGTACTCAAAAC
AAATAAACGAATGTCTAACTTTAGCCTAAAATGTGAGTT
GTCTTGTATACAAGCTTCTCGAG

PCNA

GCGGCCGCGTATACACACCTAGCCTGGTTTCCCCTAA
TTTCATTTTCCCCCTTCCATTACAATCTATTTGTTGAAG
AAATTAGATCATTTATTAAGTTTTCCAGAGTTTGGATTT
TGCTGATTGCATTCCTGTGTATACATAAATTCCTCTACC
CTGTGTGTCCTACAGACTGGTAGCTATAGCCCTGGAG
CCTTGATATTCAGGGTGTTTTGTTTCGGGGGTGAGAG
AGCAAGAATATAGGTGGTGGTGTGTGCCTCTAGTAGG
AGGCACAGGGTGTCTGGATGTGTTTGCAATGTTAGCA
GCTATAATAGTCATTGTCTAGATCCATTAAGTCATTAATT
AGAGTTTGCAGAGCTGAAATTAATACGTTTTATTCACT
TATTGGCTGCTTATTAGAAAAACTTCCATAAAGAAAAG
CTTTCCCATATTATATAATTTGGTTATCTAAATTATAGCTA
TAGGAAAGACAAGCTAGATAATCGAGTCTTTTGCTTTA
TGTATCAGTCTTCAAAATTTTCATAGCTCCCTCCAAAG
TGACCAATACAAGTGTTTGTGGGTTTTTATAAATATATA
ATGAGCTAATAGATTGCAACTTTCTTGATGTTTTTCAAT
GATGAATCTTTTGTTTTGTAGGTTACCATAGAGATGAA
TGAACCAGTTCAACTAACTTTTGCACTGAGGTACCTG
AACTTCTTTACAAAAGCCACTCCACTCTCTTCAACGG
TGACACTCAGTATGTCTGCAGATGTACCCCTTGGTAA
GATAATAAATTTGAACCTTGTTTTGTAGGTAGTCATATG
TGATACATACTCCTCAGTAATTAACCATCTTCCTGTCTT
TCAGTTGTAGAGTATAAAATTGCGGATATGGGACACTT
AAAATACTACTTGGCTCCCAAGATCGAGGATGAAGAA
GGATCTTTCGAATCTAGA

GAATTCTAGGCATTCTTAAAATTCAAGAAAATAAAACTAA
GCTCTTTGAGAACTGCTTCTAAGATGCCAGCATATACTG
AAGTCTTTTCTGTCACCAAATTTGTACCTCTAAGTACATA
TGTAGATATTGTTTTCTGTAAATAACCTATTTTTTTCTCTA
TTCTCTGCAATTTGTTTAAAGAATAAAGTCCAAAGTCAG
ATCTGGTCTAGTTAACCTAGAAGTATTTTTGTCTCTTAGA
AATACTTGTGATTTTTATAATACAAAAGGGTCTTGACTCT
AAATGCAGTTTTAAGAATTGTTTTTGAATTTAAATAAAGT
TACTTGAATTTCAAACATCACAGGGCAGTGTCTTCATTT
TGACCAGGACTGTTGGAAGTATCCTACTGAAATCCCAG
CTACAGCTACCCTTTCGTTTAAATGGTTTTTCAGTTTAG
AGCAGCCTGATGGAGATCAAAAGGATTTCAAATACAGT
AGGTTACTAGATATTTTAGGGGATTCACTTGTCAAACTT
GTTATTCCAATTTGAGCTCAAGGAACAAGTGAGAGAGT
AGTACAAATCCTGTGTATTTCATTTTACTTGGCAAAAATT
AATACTCTTCAGAAGCTTAATGTACCGAAAGTCGTGTG
GGCTTTATAAGATGTTAATGGAGTGTTTCTTTTTAGCCT
GGGGAATCTTACAAGAGTCTTGGATGTTTCAGTAGAGC
TGTAATGGGGCCCAAGGAGTATGGTTGTACAAAATTTCT
CTAGTATAAGTACACTTTGCAACTTCACTCAAAGGATGA
AGATGTCTTAAATTCCACGGATGTTGGATCCAACCAAG
AAAGTGAAAAAGCCCTGAAGTTGTGAAGAAGTATACAA
GCTTCTCGAGCAATTG
